# Validation of actigraphy sleep metrics in children aged 8 to 16 years: considerations for device type, placement and algorithms

**DOI:** 10.1186/s12966-024-01590-x

**Published:** 2024-04-16

**Authors:** K. A. Meredith-Jones, J. J. Haszard, A. Graham-DeMello, A. Campbell, T. Stewart, B. C. Galland, A. Cox, G. Kennedy, S. Duncan, R. W. Taylor

**Affiliations:** 1 Department of Medicine, University of Otago, PO Box 56, Dunedin, New Zealand; 2 Biostatistics Centre, University of Otago, Dunedin, New Zealand; 3 WellSleep Centre, University of Otago, Wellington, New Zealand; 4 School of Sport and Recreation, Auckland University of Technology, Auckland, New Zealand; 5 Fuzzy Systems Ltd, Dunedin, New Zealand; 6 Department of Women’s and Children’s Health, University of Otago, Dunedin, New Zealand

**Keywords:** Validation, Sleep/wake, Polysomnography, Algorithm, Accelerometery, Sleep

## Abstract

**Background:**

Actigraphy is often used to measure sleep in pediatric populations, despite little confirmatory evidence of the accuracy of existing sleep/wake algorithms. The aim of this study was to determine the performance of 11 sleep algorithms in relation to overnight polysomnography in children and adolescents.

**Methods:**

One hundred thirty-seven participants aged 8–16 years wore two Actigraph wGT3X-BT (wrist, waist) and three Axivity AX3 (wrist, back, thigh) accelerometers over 24-h. Gold standard measures of sleep were obtained using polysomnography (PSG; Embletta MPRPG, ST + Proxy and TX Proxy) in the home environment, overnight. Epoch by epoch comparisons of the Sadeh (two algorithms), Cole-Kripke (three algorithms), Tudor-Locke (four algorithms), Count-Scaled (CS), and HDCZA algorithms were undertaken. Mean differences from PSG values were calculated for various sleep outcomes.

**Results:**

Overall, sensitivities were high (mean ± SD: 91.8%, ± 5.6%) and specificities moderate (63.8% ± 13.8%), with the HDCZA algorithm performing the best overall in terms of specificity (87.5% ± 1.3%) and accuracy (86.4% ± 0.9%). Sleep outcome measures were more accurately measured by devices worn at the wrist than the hip, thigh or lower back, with the exception of sleep efficiency where the reverse was true. The CS algorithm provided consistently accurate measures of sleep onset: the mean (95%CI) difference at the wrist with Axivity was 2 min (-6; -14,) and the offset was 10 min (5, -19). Several algorithms provided accurate measures of sleep quantity at the wrist, showing differences with PSG of just 1–18 min a night for sleep period time and 5–22 min for total sleep time. Accuracy was generally higher for sleep efficiency than for frequency of night wakings or wake after sleep onset. The CS algorithm was more accurate at assessing sleep period time, with narrower 95% limits of agreement compared to the HDCZA (CS:-165 to 172 min; HDCZA: -212 to 250 min).

**Conclusion:**

Although the performance of existing count-based sleep algorithms varies markedly, wrist-worn devices provide more accurate measures of most sleep measures compared to other sites. Overall, the HDZCA algorithm showed the greatest accuracy, although the most appropriate algorithm depends on the sleep measure of focus.

**Supplementary Information:**

The online version contains supplementary material available at 10.1186/s12966-024-01590-x.

## Background

A large body of evidence has emerged implicating characteristics of children’s sleep such as short quantity, timing, poor quality, and high variability with a wide range of adverse health outcomes [1]. However, the majority of studies rely on retrospective self- or parent-reports of sleep, which may be unreliable and sensitive to recall bias [2, 3]. Although polysomnography (PSG) is considered the gold-standard measure of sleep, it is obtrusive and impractical for large-scale studies. Thus, actigraphy is increasingly being used as a practical and suitable method to objectively measure sleep, particularly over longer time frames than is possible with PSG. To estimate sleep outcomes, actigraphy data are analysed using algorithms to classify sleep and wake based on the assumption that the presence of movement indicates wakefulness and the absence of movement indicates sleep. Typically, algorithms vary by the population studied, device worn and the placement site they were developed for (i.e. wrist, ankle, waist), but most work in a similar fashion: to define each minute of recorded activity as either sleep or wake.

However, there are several issues with these existing algorithms. First, although various algorithms have been developed [4–9], few [7, 10] have been validated against the gold standard PSG in paediatric populations, with the remainder using sleep diaries or visual inspection. Second, choice of algorithm influences sleep–wake time estimates suggesting that sleep variables derived from different algorithms might not be comparable [11]. Third, although currently available sleep algorithms provide reasonable estimates of sleep, most require participants to record their sleep onset and waking times, which are used to guide the algorithm to detect nocturnal sustained bouts of inactivity. However, sleep diaries are often inaccurate, add to participant burden, and are time consuming for researchers in large scale studies [12]. To overcome these limitations, fully automated algorithms that do not require diaries have been developed for use in children which automatically score sleep [5–9] but evidence of their accuracy against PSG is limited [10, 13].

With the growing availability of accelerometry data from large studies, often without sleep diaries, it is necessary to establish whether sleep outcomes are comparable between brands and across various wear sites. It is also important to evaluate sleep outcome estimates between the most widely used sleep–wake algorithms, with and without the use of sleep diaries to guide the algorithm. Therefore, the aim of this study is to compare the accuracy of the most widely used sleep algorithms against overnight PSG in children and adolescents.

## Methods

### Participants

Children and adolescents were recruited via social media (i.e. Facebook), schools, and word of mouth. Children aged 8 to 16 years at the time of recruitment with no history of sleep disturbance (see below) were eligible for the study. Ethical approval was obtained from the University of Otago Human Ethics Committee (ref H18/073).

### Data collection overview

During a visit to each participant’s home height and weight were measured and five accelerometers were attached to the child (two on the wrist, one around their waist, one on their lower back, and one on their upper thigh). These devices were worn for one 24-h period. Participants were also fitted with a portable polysomnography (PSG) machine one hour before bedtime to measure sleep during the overnight period in the home environment. Children were asked to complete a basic activity log the next day. The same computer was used to program the accelerometers and the PSG recording device and times were synchronized.

### Sleep Disturbances Scale for Children (SDSC)

Parents completed the SDSC consisting of 27 items assessing sleep behaviour and disturbances in children in the previous six months [14]. A total sleep problem score is derived from six sleep disturbance factors. A score greater than 39 is indicative of a clinical disturbance and those identified as having a sleep disorder, or those with any chronic medical condition or physical disability that impeded their ability to participate in physical activity, were excluded.

### Demographic and anthropometric data

Information was collected on participant’s age, sex, date of birth, and ethnicity using New Zealand census questions [15]. Their address was used to determine area based socio-economic status using the New Zealand Deprivation Index (NZDep Index, 2018) [16]. Duplicate measures of height (Model 213, Seca, Germany) and weight (Tanita HD-351) were obtained by trained research assistants. An additional measure was undertaken if duplicate measures of height differed by more than 0.5 cm and if weight differed by more than 0.5 kg. Body mass index (BMI) was calculated as weight (kg) / height (m)^2^, with overweight and obesity defined as a BMI z-score ≥ 85^th^ but < 95^th^ and ≥ 95^th^ percentiles, respectively, using the WHO growth reference [17].

### Home-based polysomnography

A home-based, PSG sleep study was conducted where overnight PSG data were recorded using a digital portable monitor (Embletta MPRPG, ST + Proxy and TX Proxy, Natus, California, USA) within participant’s homes at a sampling rate of 500 Hz following American Academy of Sleep Medicine guidelines [18]. The researcher began the PSG set up approximately one hour before bedtime. The PSG included right and left electro-oculograms (EOG), four electroencephalograms (EEG) (C4/M1, C3/M2, O2/M1, O1/M2), chin electromyogram, nasal airflow, snoring, thoracic and abdominal respiratory effort (Xact Trace Respiratory Effort Sensor) and ECG. Oxygen saturation was measured with pulse oximetry. Data were downloaded and analysed using RemLogic software (Version 3.4, Embla Systems, Broomfield, CO, USA). Low frequency filters were set at 0.3 Hz and high frequency at 35 Hz for EEG signals. Sleep stages were scored visually by one trained sleep technician in 30 s epochs using the American Academy of Sleep Medicine (AASM) sleep staging criteria [18] for children. To allow for comparison to actigraphy, the PSG epoch lengths were collapsed into one-minute epochs. In doing so, if either 30-s epoch within the minute was scored as wake, then we considered that whole minute as wake. For PSG, sleep onset was the first epoch of sleep after lights out. Total sleep time (TST) was defined as the number of minutes from sleep onset to sleep offset minus the number of minutes awake. Wake after sleep onset (WASO) represented the duration of time spent awake after initially falling asleep, while sleep efficiency (SE) was defined as follows: 1) Sleep efficiency_TIB_, a commonly referenced metric, calculated as the ratio of total sleep time (TST) to time spent in bed (TIB); and 2) Sleep efficiency_SPT_, determined by expressing total sleep time (from sleep onset to offset, minus any WASO) as a percentage of sleep period time (from sleep onset to offset, inclusive of any WASO). We chose to use the Sleep Period Time (SPT) in our definition of Sleep efficiency_SPT_ alongside the more traditional definition which uses TIB because one of our aims was to compare the accuracy of algorithms that required sleep diaries versus those that did not. Furthermore, the definition of SE that uses TIB, by definition, includes non-sleep related activity (eg reading, texting, mobile phone use) both prior to initiating sleep and after the final awakening, which do not reflect the construct of SE where TST is compared to the amount of time spent attempting to initially fall asleep and sleep discontinuity. Number of awakenings was the number of overnight awakenings between sleep onset to offset. The PSG and actigraphy data were analysed independently by different researchers.

### Actigraphy

Two types of accelerometers were worn: the Axivity AX3 (Axivity Ltd, Newcastle, UK), and the Actigraph wGT3X-BT (ActiGraph, Pensacola, FL, USA). Both accelerometers are triaxial and were configured to record at a frequency of 100 Hz and initialised using the same personal computer as the PSG. The compact size (32.5 × 23 × 8.9 mm), lightweight design (11 g), and waterproof feature of the Axivity AX3s contribute to higher compliance among children, while the inclusion of a temperature sensor assists in non-wear detection. The Actigraph wGT3X-BT is currently the most widely used research-grade device and is larger (46 × 33 x 15 mm, 19 g) than the Axivity AX3 and lacks a temperature sensor. The three Axivity accelerometers were fitted to the right side of the lower back (waist-level), middle of the right thigh, and non-dominant wrist using custom designed hypoallergenic tape. Two Actigraph accelerometers were fitted to participants at two main sites: the non-dominant wrist using an elastic wrist strap and over the right hip using custom designed hypoallergenic tape. Axivity devices were set up and data downloaded with OmGui software version 1.0.0.30 (Open Movement, Newcastle, UK). ActiGraph wGT3X-BT devices were initialised and downloaded using ActiLife version 6.13.3, saved in raw format as.gt3x, then converted for data processing. Raw acceleration data from the Actigraph and Axivity were processed and calibrated using the open-access Pampro package v0.5 [19] and converted into hdf5 file formats for processing. All algorithms except the HDCZA were written in the Python programming language (Python Software Foundation, https://www.python.org/) and outputs were computed using this same software system, rather than proprietary device software. Data analysed using the HDCZA algorithm were processed and analysed with R-package GGIR version 1.2–0 (http://cran.r-project.org) [20].

### Algorithms

The selection of algorithms featured in this manuscript was informed by a comprehensive review of pertinent literature pertaining to prevalent methodologies utilized for estimating sleep patterns in pediatric populations employing count-based actigraphy. Additionally, consideration was given to algorithms integrated within the proprietary software accompanying the Actigraph GT3X + devices. Details of how each algorithm scores sleep and wake and calculates each sleep outcome are given in Table 1. Briefly, we included three versions of the Cole-Kripke algorithm [5], two versions of the Sadeh algorithm [13], four versions of the Tudor-Locke algorithm [4, 8], the count-scaled (CS) algorithm [6], and the HDCZA algorithm [9]. In general, the versions of each algorithm differed mostly by whether they required the use of diaries to estimate sleep onset and offset and whether they included variations to account for changes in sensitivity between older and newer accelerometer models.

**Table 1:** Scoring for each algorithm

Algorithm	Sleep/wake epochs scored as 1 or 0 based on	Parameter algorithm used
Count-scaled	Count-scaled algorithm [6]	Uses ‘average’ estimated bedtime and waketime for population under study. To detect the bedtime sleep “event” the algorithm first moves 3 h forward to detect the first sleep onset event. If sleep is not detected in this 3 h it moves 2 h backwards to identify the last sleep onset event. If a sleep event is not detected within the 3 h after or 2 h before the chosen bedtime, the chosen bedtime (e.g. 7:30 pm) is used. Sleep onset = first of 15 continuous minutes of sleep preceded by 5 min of awake. Sleep offset: last of 15 continuous minutes of sleep followed by 5 min of awake. Awakening: 5 continuous minutes of awake preceded and followed by 15 min of sleep.
Sadeh 1	Sadeh algorithm [13] used in Actilife that changes the algorithm sleep wake thresholds based on newer model sensitivities	This uses the original Sadeh algorithm with changes to incorporate the sensitivity differences between the original accelerometer used to develop the algorithm and newer models. The Sadeh algorithm uses an 11-min window that includes the five previous and five future epochs. Note: any missing epochs are considered ZERO. This happens if the current epoch is at the beginning or end of a dataset. The Sadeh algorithm uses the y-axis epoch data. If any of the epoch counts are over 300, it reduces them to 300. The algorithm requires the following information about the window that you’re looking at: Arithmetic mean (average) of the activity counts for the window (AVG) Number of epochs that have counts ≥ 50 and < 100 (NATS) Standard deviation for the first 6 epochs of the window (SD) Natural (base e) logarithm of a current epoch. Note: If the epoch count is 0, we make this value 0 to avoid infinity problems (LG). Those calculations are put through the following algorithm: (7.601—(0.065 * AVG)—(1.08 * NATS)—(0.056 * SD)—(0.703 * LG)). If the result of that algorithm is GREATER than -4, then current epoch is considered sleep. The original algorithm used a > 0 threshold and Actigraph changed this to -4 because of the differences between old and new models. Requires diary times for TIB and TOB.
Sadeh 2	Sadeh algorithm [13] used in Actilife that changes the algorithm sleep wake thresholds based on newer model sensitivities	Same as above, except entirely automated so diaries are not used
Cole-Kripke 1	Original Cole-Kripke algorithm [5]	The Cole-Kripke algorithm computes a weighted sum of the activity in the current minute, the preceding 4 min, and the following 2 min as follows: S = 0.0033(1.06an4 + 0.54an3 + 0.58an2 + 0.76an1 + 2.3a0 + 0.74al + 0.67a2) where an4–an1 are activity counts from the prior 4 min, a0 is the current minute, and a1 and a2 are the following 2 min. The current minute is scored as sleep when S < 1. Includes the Webster rescoring rules: (a) After at least 4 min scored as wake, the next 1 min scored as sleep is rescored as wake; (b) after at least 10 min scored as wake, the next 3 min scored as sleep are rescored as wake; (c) after at least 15 min scored as wake, the next 4 min scored as sleep are rescored as wake; (d) 6 min or less scored as sleep surrounded by at least 10 min (before and after) scored as wake are rescored as wake.
Cole-Kripke 2	Based on Cole-Kripke algorithm that is used in Actilife software and uses diary sleep and wake times	Same as above but the epoch data are adjusted to help reduce the variation of the counts from the original devices compared to newer actigraphs. Using the y-axis epoch data, counts are divided by 100. If any of those scaled values are over 300, set them to 300. Also uses diary times to constrain the algorithm.
Cole-Kripke 3	Based on Cole-Kripke algorithm that is used in Actilife software, but does not use the diary sleep and wake times	Same as above but does not use diary times to constrain the algorithm.
Tudor-Locke 1	Based on Cole-Kripke algorithm that is used in Actilife software and uses diary sleep and wake times	Original Tudor-Locke automated method [8] that rescores the sleep/wake states using inclinometer data. Bedtime is identified as the first 5 consecutive minutes defined as sleep. Similarly, wake time is identified as the first 10 consecutive minutes defined as wake after a period of sleep. Bedtime and wake time are only identified when at least 160 min has elapsed between these 2 time points. An unlimited number of nonconsecutive wake minutes are allowed between bedtime and wake time, in keeping with the definition of sleep-period time that includes all sleep epochs and wakefulness after onset. Multiple sleep periods (≥ 160 min) are allowed during each 24-h day. The algorithm was constructed to output the beginning and ending minutes for each sleep period identified, but ultimately retains only the beginning minute of the first period (bedtime) in the time block studied and the final minute of the last period (wake time). Sleep-period time is ultimately calculated as the number of minutes between bedtime and wake time. Does not account for the possibility of nocturnal nonwear or extended episodes of wakefulness separating the SPT into multiple sleep episodes. Also does not consider the potential for misclassifying daytime periods of nonwear or other sedentary behaviors as sleep episodes (i.e., “naps”).
Tudor-Locke 2	Based on Cole-Kripke algorithm that is used in Actilife software and uses diary sleep and wake times	Refined Tudor Locke method [4] rescores sleep/wake states using the inclinometer to identify the probability of sleep and define parameters. Constrains algorithm to nocturnal sleep, by rule that only allows sleep onset between 7:00 p.m. and 5:59 a.m. Sleep offset refined and identified as the first of 10 or 20 consecutive inclinometer revised scored wake minutes, depending on the time of day (10 min—5:00 a.m. to 11:58 a.m.; 20 min—9:40 p.m. to 4:59 a.m.). The RSA allows identification of extended episodes of wakefulness that separate the sleep period time into distinct sleep episodes with multiple sleep onsets and offsets. If two sleep episodes were separated by less than 20 min of inclinometer re-scored wake minutes, then they were combined into a single sleep episode starting with the first minute of the first sleep episode and ending with the final minute of the last sleep episode. Sleep episodes that were separated by at least 20 min of inclinometer re-scored wake minutes were distinct within the sleep period time and were not combined. Total sleep episode time (TSET) represented the total minutes from all sleep episodes; in cases where there was a single sleep episode, or all sleep episodes were separated by less than 20 min of inclinometer re-scored wake, the TSET was equal to RSA sleep period time. Nonwear was identified when 90 consecutive minutes of 0 activity counts were encountered while allowing for up to 2 min of nonzero activity counts. The nonwear period ended when a third minute of nonzero activity counts was detected. If at least 90% of a sleep episode was categorized as nonwear, then all minutes within that sleep episode were redefined as nonwear and not included in the calculation of TSET.
Tudor-Locke 3	Sadeh algorithm [13] used in Actilife that changes the algorithm sleep wake thresholds based on newer model sensitivities	Original Tudor-Locke automated method [8] that rescores the sleep/wake states using inclinometer data. Bedtime is identified as the first 5 consecutive minutes defined as sleep. Similarly, wake time is identified as the first 10 consecutive minutes defined as wake after a period of sleep. Bedtime and wake time are only identified when at least 160 min has elapsed between these 2 time points. An unlimited number of nonconsecutive wake minutes are allowed between bedtime and wake time, in keeping with the definition of sleep-period time that includes all sleep epochs and wakefulness after onset. Multiple sleep periods (≥ 160 min) are allowed during each 24-h day. The algorithm was constructed to output the beginning and ending minutes for each sleep period identified, but ultimately retains only the beginning minute of the first period (bedtime) in the time block studied and the final minute of the last period (wake time). Sleep-period time is ultimately calculated as the number of minutes between bedtime and wake time. Does not account for the possibility of nocturnal nonwear or extended episodes of wakefulness separating the SPT into multiple sleep episodes. Also does not consider the potential for misclassifying daytime periods of nonwear or other sedentary behaviors as sleep episodes (i.e., “naps”).
Tudor-Locke 4	Sadeh algorithm [13] used in Actilife that changes the algorithm sleep wake thresholds based on newer model sensitivities	Refined Tudor Locke method [4] rescores sleep/wake states using the inclinometer to identify the probability of sleep and define parameters. Constrains algorithm to nocturnal sleep, by rule that only allows sleep onset between 7:00 p.m. and 5:59 a.m. Sleep offset refined and identified as the first of 10 or 20 consecutive inclinometer revised scored wake minutes, depending on the time of day (10 min—5:00 a.m. to 11:58 a.m.; 20 min—9:40 p.m. to 4:59 a.m.). The RSA allows identification of extended episodes of wakefulness that separate the sleep period time into distinct sleep episodes with multiple sleep onsets and offsets. If two sleep episodes were separated by less than 20 min of inclinometer re-scored wake minutes, then they were combined into a single sleep episode starting with the first minute of the first sleep episode and ending with the final minute of the last sleep episode. Sleep episodes that were separated by at least 20 min of inclinometer re-scored wake minutes were distinct within the sleep period time and were not combined. Total sleep episode time (TSET) represented the total minutes from all sleep episodes; in cases where there was a single sleep episode, or all sleep episodes were separated by less than 20 min of inclinometer re-scored wake, the TSET was equal to RSA sleep period time. Nonwear was identified when 90 consecutive minutes of 0 activity counts were encountered while allowing for up to 2 min of nonzero activity counts. The nonwear period ended when a third minute of nonzero activity counts was detected. If at least 90% of a sleep episode was categorized as nonwear, then all minutes within that sleep episode were redefined as nonwear and not included in the calculation of TSET.
HDCZA	[9]	Calculates wrist rotation (changes in the z-angle) for each 5-min rolling window and values under the 10th percentile over an individual day (noon-to-noon). The algorithm then detects blocks lasting > 30 min, with gaps < 60 min counted towards the identified blocks. The longest block in the day between noon–noon represents the sleep period window. Sleep episodes were defined as the sustained periods of inactivity within the sleep period window. From this, the number of sleep episodes within each sleep period window detected is calculated as well as sleep efficiency within the sleep period window calculated as the percentage of time asleep within the sleep period. Note, newer versions of this algorithm can use values under the 13th, 20th and 50th percentile.

### Statistical analyses

#### Epoch-by epoch comparison

One-minute epochs from the Axivity thigh, wrist, and lower back and Actigraph waist and wrist were aligned with corresponding PSG epochs. Agreement between the Axivity and Actigraph at each site placement (wrist, thigh, lower back, waist) and PSG (as the gold standard) were examined by calculating overall agreement (%), sensitivity (% sleep agreement), and specificity (% wake agreement).

Sleep outcomes were organised into three categories: sleep timing (sleep onset and offset), sleep quantity (sleep period time and total sleep time), and sleep quality (WASO, sleep efficiency, and number of night wakings). These were described with means and standard deviations and compared to PSG by calculating the mean difference and 95% confidence interval. Only participants with data for all outcomes were included for each device and placement.

Bland Altman plots were used to explore agreement against PSG for the “*overall* best performing” algorithm, regardless of placement site or device (by % accuracy) and for the “best performing algorithm” (by mean difference from PSG) for the site placement and device deemed to be the best performing for SPT (a measure dependent on sleep onset and sleep offset and not dependent on WASO) and WASO. Mean differences and 95% limits of agreement were calculated. Stata 17.0 (StataCorp, Texas) was used for all analyses.

## Results

### Study participants

In total, 384 children completed the screening questionnaire. Of these, 202 were ineligible, due to age (*n* = 4), lived outside the Dunedin area (*n* = 12) or had a sleep disturbance score greater than 39 (*n* = 186). A total of 182 participants were eligible to participate and of these 151 expressed further interest in the study. PSG was conducted in 138 participants with early termination of PSG for one participant due to technical failure, leaving 137 participants included in the final analyses (Supplementary Table 1 for details on missing data). The characteristics of the participants are shown in Table 2. The majority of participants were of New Zealand European ethnicity, slightly more boys participated than girls, and 37% of the sample were overweight or obese.

**Table 2:** Characteristics of the study population

		Males	Females	Total
n		70	67	137
Age (years)		11.6 (2.1)	10.8 (2.3)	11.2 (2.3)
Ethnicity, n (%)	New Zealand European & Others	59 (84%)	49 (73%)	108 (79%)
	Māori	9 (13%)	13 (19%)	22 (16%)
	Pacific	2 (3%)	5 (8%)	7 (5%)
Household deprivation^a^, n (%)	Low	35 (50%)	29 (43%)	64 (47%)
	Medium	23 (33%)	23 (34%)	46 (34%)
	High	12 (17%)	15 (22%)	27 (20%)
Height (cm)		153.0 (14.2)	148.0 (14.2)	150.5 (14.4)
Weight (kg)		46.4 (13.7)	45.8 (18.0)	46.1 (15.8)
Body mass index (BMI, kg/m^2^)		19.4 (3.2)	20.2 (4.9)	19.8 (4.1)
Weight status^b^, n (%)	Normal weight	48 (69%)	39 (58%)	87 (64%)
	Overweight	22 (31%)	28 (42%)	50 (37%)

Data presented as mean (SD) except where noted

^a^Uses the New Zealand Index of Deprivation 2013, which reflects the extent of material and social deprivation and is used to construct deciles from 1 (least deprived) to 10 (most deprived) [16]

^b^Categories based on the WHO BMI z-score cut-offs [17]

## Epoch by epoch analyses

### Placement and device

#### Actigraph vs Axivity at the wrist vs waist, lower back, thigh

Table 3 demonstrates that in general, overall accuracy tended to be higher for both devices placed at the wrist (mostly greater than 80%) than when placed close to the centre of mass (waist, thigh, and lower back, where accuracy was generally less than 80%). However, different patterns were observed for sensitivity and specificity. Sensitivity, or the ability to detect episodes of sleep was generally higher when placed closer to the centre of mass for both the Actigraph and Axivity compared to the wrist. By contrast, specificity (% wake agreement) was considerably better for both devices at the wrist than at the waist.

**Table 3:** Sensitivity, specificity, and accuracy of epoch-by-epoch comparisons with PSG for sleep

Device	Placement	Algorithm	Mean accuracy % (95% CI)	Mean sensitivity % (95% CI)	Mean specificity % (95% CI)
Actigraph GT3x	Hip	Count-scaled	77.6 (75.7, 79.5)	95.3 (94.0, 96.6)	56.8 (53.8, 59.7)
		Sadeh 1	78.6 (76.6, 80.6)	97.7 (96.4, 99.0)	55.9 (52.7, 59.1)
		Sadeh 2	79.0 (76.9, 81.0)	97.6 (96.3, 99.0)	56.5 (53.3, 59.7)
		Cole-Kripke 1	82.0 (79.9, 84.1)	94.3 (92.6, 96.0)	67.8 (64.4, 71.1)
		Cole-Kripke 2	73.1 (71.3, 75.0)	98.8 (97.7, 99.8)	42.4 (39.7, 45.1)
		Cole-Kripke 3	73.5 (71.6, 75.4)	98.7 (97.6, 99.8)	43.2 (40.4, 46.0)
		Tudor-Locke 1	73.7 (71.8, 75.6)	98.8 (97.6, 99.9)	43.1 (40.3, 45.8)
		Tudor-Locke 2	72.9 (71.0, 74.8)	98.8 (97.7, 99.9)	42.0 (39.2, 44.7)
		Tudor-Locke 3	78.4 (76.4, 80.4)	98.0 (96.7, 99.3)	54.8 (51.5, 58.0)
		Tudor-Locke 4	78.6 (76.7, 80.6)	98.0 (96.7, 99.3)	54.9 (51.7, 58.1)
		HDCZA	85.4 (83.3, 87.6)	85.8 (82.9, 88.6)	85.9 (83.7, 88.0)
	Wrist	Count-scaled	82.3 (80.3, 84.2)	93.2 (91.5, 94.8)	69.7 (66.6, 72.9)
		Sadeh 1	84.1 (82.1, 86.1)	90.9 (89.0, 92.7)	76.4 (73.2, 79.6)
		Sadeh 2	84.4 (82.4, 86.4)	90.9 (89.1, 92.8)	76.9 (73.8, 80.1)
		Cole-Kripke 1	79.8 (77.9, 81.7)	77.9 (75.7, 80.1)	82.8 (79.8, 85.9)
		Cole-Kripke 2	83.7 (81.7, 85.8)	94.4 (92.7, 96.1)	71.3 (68.0, 74.7)
		Cole-Kripke 3	84.4 (82.5, 86.3)	94.9 (93.6, 96.2)	72.1 (68.8, 75.4)
		Tudor-Locke 1	83.5 (81.4, 85.5)	95.1 (93.4, 96.8)	69.7 (66.4, 73.1)
		Tudor-Locke 2	84.0 (82.1, 85.9)	95.0 (93.3, 96.7)	70.4 (67.2, 73.6)
		Tudor-Locke 3	83.9 (81.9, 85.9)	92.4 (90.6, 94.2)	74.1 (70.9, 77.3)
		Tudor-Locke 4	84.1 (82.1, 86.1)	92.4 (90.6, 94.2)	74.4 (71.2, 77.5)
		HDCZA	86.1 (84.2, 88.1)	85.6 (83.2, 88.0)	87.5 (85.4, 89.7)
Axivity	Back	Count-scaled	74.3 (72.1, 76.4)	92.3 (90.6, 93.9)	52.8 (49.4, 56.2)
		Sadeh 1	75.4 (73.2, 77.7)	95.4 (93.7, 97.1)	51.8 (48.4, 55.3)
		Sadeh 2	76.5 (74.3, 78.7)	95.8 (94.3, 97.3)	53.6 (50.2, 56.9)
		Cole-Kripke 1	78.7 (76.4, 81.0)	92.7 (90.8, 94.5)	62.6 (58.9, 66.2)
		Cole-Kripke 2	71.6 (69.5, 73.6)	97.0 (95.5, 98.4)	41.2 (38.2, 44.1)
		Cole-Kripke 3	72.4 (70.3, 74.4)	97.3 (96.0, 98.6)	42.5 (39.6, 45.5)
		Tudor-Locke 1	72.3 (70.2, 74.3)	97.3 (96.0, 98.6)	42.2 (39.2, 45.2)
		Tudor-Locke 2	72.1 (70.1, 74.1)	97.5 (96.3, 98.7)	41.7 (38.7, 44.7)
		Tudor-Locke 3	76.0 (73.7, 78.3)	95.3 (93.5, 97.0)	53.2 (49.8, 56.5)
		Tudor-Locke 4	76.2 (74.0, 78.4)	95.9 (95.5, 97.4)	53.1 (49.7, 56.5)
		HDCZA	85.8 (83.6, 88.0)	87.7 (85.5, 90.0)	87.1 (85.1, 89.1)
	Thigh	Count-scaled	71.9 (69.7, 74.1)	88.1 (86.1, 90.1)	52.8 (49.8, 55.7)
		Sadeh 1	77.6 (75.2, 80.1)	90.6 (88.1, 93.1)	62.3 (58.7, 65.8)
		Sadeh 2	77.9 (75.5, 80.4)	91.0 (88.6, 93.4)	62.5 (59.0, 66.1)
		Cole-Kripke 1	78.4 (75.9, 80.9)	84.4 (81.7, 87.1)	71.3 (67.6, 74.9)
		Cole-Kripke 2	75.0 (72.6, 77.3)	94.0 (91.8, 96.2)	52.4 (49.2, 55.6)
		Cole-Kripke 3	75.7 (73.3, 78.0)	94.3 (92.1, 96.4)	53.8 (50.6, 56.9)
		Tudor-Locke 1	75.1 (72.7, 77.5)	94.0 (91.8, 96.2)	52.8 (49.7, 56.0)
		Tudor-Locke 2	76.2 (74.0, 78.3)	95.5 (94.1, 96.9)	53.5 (50.3, 56.7)
		Tudor-Locke 3	77.6 (75.1, 80.1)	90.5 (88.0, 93.1)	62.2 (58.7, 65.8)
		Tudor-Locke 4	78.7 (76.4, 81.0)	91.6 (89.6, 93.7)	63.3 (59.8, 66.7)
		HDCZA	87.3 (85.5, 89.0)	88.5 (87.0, 90.0)	87.2 (85.1, 89.3)
	Wrist	Count-scaled	77.4 (75.1, 79.7)	86.0 (83.6, 88.3)	67.3 (63.9, 70.7)
		Sadeh 1	79.8 (77.4, 82.2)	83.9 (81.4, 86.5)	75.6 (72.2, 78.9)
		Sadeh 2	80.3 (78.0, 82.6)	84.5 (82.1, 86.9)	76.0 (72.6, 65.8)
		Cole-Kripke 1	76.0 (73.8, 78.2)	72.6 (70.3, 74.9)	81.0 (77.8, 84.2)
		Cole-Kripke 2	80.0 (77.5, 82.4)	87.7 (85.0, 90.4)	70.8 (67.2, 74.3)
		Cole-Kripke 3	80.1 (77.7, 82.5)	87.6 (85.0, 90.3)	71.1 (67.5, 74.6)
		Tudor-Locke 1	80.0 (77.5, 82.4)	87.7 (85.0, 90.4)	70.8 (67.2, 74.3)
		Tudor-Locke 2	80.5 (78.2, 82.8)	88.9 (86.7, 91.0)	70.7 (67.1, 74.3)
		Tudor-Locke 3	80.3 (78.0, 82.6)	84.5 (82.1, 86.9)	76.0 (72.6, 79.3)
		Tudor-Locke 4	80.1 (77.7, 82.4)	84.5 (82.2, 86.8)	75.5 (72.1, 78.9)
		HDCZA	87.5 (85.7, 89.4)	87.5 (85.9, 89.0)	89.6 (87.8, 91.3)

#### Algorithms vs placement

Site of placement did not appear to affect the overall accuracy or sensitivity for each algorithm to a great extent as most algorithms appeared to perform similarly when placed close to the centre of mass (thigh, lower back, waist) or at the wrist, varying by less than 10% (Table 3). However, site of placement had a large effect on specificity for most algorithms with only the HDCZA algorithm varying by less than 10% between placements. Regardless of placement, we report similar total accuracy across the HDCZA, CS, Sadeh 1, Sadeh 2, Cole-Kripke 1, Tudor-Locke 3, and Tudor-Locke 4 algorithms, but lower accuracy for the Cole-Kripke 2, Cole-Kripke 3, Tudor-Locke 1, and Tudor-Locke 2 algorithms. Given the difficulty of actigraphy to detect periods of wakefulness during sleep, the considerably higher level of specificity for the HDCZA algorithm (ranging from 85.9% to 89.6%), compared to all others which showed specificities as low as 41.2%, with many less than 60%, should be noted.

A sensitivity analysis (Supplementary Table 2) was undertaken to determine the effect of the post-processing merge of PSG epochs into 60-s. In the original analyses if either 30-s epoch within the minute was scored as wake, we considered that whole minute as wake, whereas in the sensitivity analyses if either 30-s epoch within the minute was scored as sleep, we considered that whole minute as sleep. For most algorithms (apart from a few placed at the wrist) this resulted in marginal increases in accuracy (< 2%) as a result of increases in specificity (the ability to detect wake-time) at the expense of decreases in sensitivity (the ability to detect sleep time).

## Sleep outcomes

Tables 4 (Actigraph) and 5 (Axivity) report differences between each algorithm and PSG for relevant sleep outcomes of interest in three broad categories: sleep timing (sleep onset and offset), sleep quantity (sleep period time and total sleep time), and sleep quality (sleep efficiency, WASO, and number of night wakings).

**Table 4:** Comparison of PSG and Actigraph GT3x measured sleep outcomes using different algorithms and at each site

Sleep variable	Device	Placement	Algorithm	Mean (SD mins)	Mean difference (95% CI) from PSG^a^
Sleep onset (hh:mm)	PSG	*n* = 114		21:38 (58)	Reference
	Actigraph GT3x	Hip (*n* = 114)	Count-scaled	21:26 (61)	-12 (-20, -4)
			Sadeh 1	21:23 (54)	-15 (-20, -10)
			Sadeh 2	20:39 (112)	-59 (-79, -39)
			Cole-Kripke 1	21:31 (53)	-7 (-12, -2)
			Cole-Kripke 2	21:20 (51)	-18 (-23, -14)
			Cole-Kripke 3	19:10 (153)	-149 (-179, -119)
			Tudor-Locke 1	19:49 (127)	-109 (-132, -87)
			Tudor-Locke 2	19:47 (49)	-112 (-124, -100)
			Tudor-Locke 3	20:48 (97)	-50 (-67, -34)
			Tudor-Locke 4	20:24 (64)	-74 (-85, -63)
			HDCZA	**21:33 (75)**	**-6 (-15, 4)**
	PSG	*n* = 119		21:37 (57)	Reference
	Actigraph GT3x	Wrist (*n* = 119)	Count-scaled	**21:39 (57)**	**2 (-3, 7)**
			Sadeh 1	**21:33 (54)**	**-4 (-8, 1)**
			Sadeh 2	**21:22 (120)**	**-15 (-37, 6)**
			Cole-Kripke 1	21:46 (60)	8 (3, 13)
			Cole-Kripke 2	21:31 (53)	-6 (-12, -1)
			Cole-Kripke 3	21:05 (114)	-32 (-52, -11)
			Tudor-Locke 1	21:01 (112)	-37 (-57, -16)
			Tudor-Locke 2	21:07 (57)	-30 (-38, -22)
			Tudor-Locke 3	**21:37 (121)**	**-20 (-42, 2)**
			Tudor-Locke 4	21:24 (58)	-13 (-18, -8)
			HDCZA	21:33 (58)	-4 (-8, -1)
Sleep offset (hh:mm)	PSG	*n* = 114		6:44 (90)	Reference
	Actigraph GT3x	Hip (*n* = 114)	Count-scaled	7:00 (63)	16 (1, 31)
			Sadeh 1	6:58 (57)	14 (1, 28)
			Sadeh 2	7:36 (86)	53 (34, 71)
			Cole-Kripke 1	6:57 (56)	13 (0, 27)
			Cole-Kripke 2	6:59 (56)	15 (1, 29)
			Cole-Kripke 3	8:17 (92)	93 (73, 114)
			Tudor-Locke 1	8:03 (88)	79 (60, 98)
			Tudor-Locke 2	7:58 (83)	74 (56, 93)
			Tudor-Locke 3	7:34 (83)	51 (33, 68)
			Tudor-Locke 4	7:29 (76)	46 (29, 62)
			HDCZA	**6:33 (141)**	**-11 (-40, 18)**
	PSG	*n* = 119		6:43 (91)	Reference
	Actigraph GT3x	Wrist (*n* = 119)	Count-scaled	**6:53 (62)**	**9 (-5, 24)**
			Sadeh 1	**6:52 (61)**	**9 (-4, 22)**
			Sadeh 2	**6:37 (93)**	**-6 (-26, 14)**
			Cole-Kripke 1	**6:53 (60)**	**10 (-4, 23)**
			Cole-Kripke 2	**6:55 (61)**	**12 (-1, 26)**
			Cole-Kripke 3	**6:56 (92)**	**12 (-8, 33)**
			Tudor-Locke 1	**7:00 (89)**	**16 (-4, 36)**
			Tudor-Locke 2	7:04 (62)	21 (6, 36)
			Tudor-Locke 3	**6:55 (79)**	**12 (-6, 29)**
			Tudor-Locke 4	**6:56 (63)**	**12 (-4, 28)**
			HDCZA	**7:00 (95)**	**16 (-2, 35)**
Sleep period time (min)	PSG	*n* = 114		545 (92)	Reference
	Actigraph GT3x	Hip (*n* = 114)	Count-scaled	573 (75)	28 (13, 43)
			Sadeh 1	575 (61)	29 (15, 44)
			Sadeh 2	658 (142)	113 (84, 142)
			Cole-Kripke 1	566 (59)	20 (6, 35)
			Cole-Kripke 2	579 (60)	34 (20, 47)
			Cole-Kripke 3	788 (201)	243 (203, 283)
			Tudor-Locke 1	734 (174)	189 (156, 222)
			Tudor-Locke 2	732 (90)	187 (167, 207)
			Tudor-Locke 3	647 (121)	102 (78, 126)
			Tudor-Locke 4	666 (87)	121 (102, 140)
			HDCZA	**553 (104)**	**8 (-15, 31)**
	PSG	*n* = 119		546 (93)	Reference
	Actigraph GT3x	Wrist (*n* = 119)	Count-scaled	**553 (66)**	**7 (-7, 21)**
			Sadeh 1	**559 (66)**	**13 (-1, 26)**
			Sadeh 2	**556 (151)**	**10 (-22, 42)**
			Cole-Kripke 1	**548 (60)**	**1 (-12, 15)**
			Cole-Kripke 2	565 (66)	19 (6, 31)
			Cole-Kripke 3	591 (137)	45 (17, 73)
			Tudor-Locke 1	600 (136)	54 (27, 81)
			Tudor-Locke 2	598 (67)	52 (36, 68)
			Tudor-Locke 3	579 (145)	33 (2, 63)
			Tudor-Locke 4	572 (66)	26 (10, 43)
			HDCZA	567 (92)	21 (2, 40)
Total sleep time (min)	PSG	*n* = 114		518 (89)	Reference
	Actigraph GT3x	Hip (*n* = 114)	Count-scaled	**532 (73)**	**14 (-1, 29)**
			Sadeh 1	557 (59)	39 (25, 54)
			Sadeh 2	623 (118)	105 (80, 130)
			Cole-Kripke 1	**527 (60)**	**9 (-6, 24)**
			Cole-Kripke 2	571 (59)	53 (39, 67)
			Cole-Kripke 3	734 (158)	216 (183, 248)
			Tudor-Locke 1	701 (148)	183 (154, 212)
			Tudor-Locke 2	695 (86)	177 (157, 196)
			Tudor-Locke 3	619 (109)	101 (78, 123)
			Tudor-Locke 4	628 (81)	110 (92, 128)
			HDCZA	495 (102)	-23 (-45, -2)
	PSG	*n* = 119		519 (89)	Reference
	Actigraph GT3x	Wrist (*n* = 119)	Count-scaled	502 (61)	-16 (-30, -2)
			Sadeh 1	496 (58)	-22 (-36, -9)
			Sadeh 2	490 (105)	-29 (-53, -4)
			Cole-Kripke 1	418 (55)	-101 (-117, -84)
			Cole-Kripke 2	**523 (61)**	**5 (-8, 18)**
			Cole-Kripke 3	**540 (97)**	**21 (-1, 43)**
			Tudor-Locke 1	550 (96)	32 (11, 53)
			Tudor-Locke 2	549 (60)	31 (15, 46)
			Tudor-Locke 3	**514 (102)**	**-5 (-28, 19)**
			Tudor-Locke 4	**514 (60)**	**-5 (-20, 11)**
			HDCZA	486 (91)	-33 (-51, -15)
WASO (min)	PSG	*n* = 114		74 (126)	Reference
	Actigraph GT3x	Hip (*n* = 114)	Count-scaled	41 (18)	-33 (-56, -9)
			Sadeh 1	17 (17)	-56 (-79, -34)
			Sadeh 2	35 (37)	-39 (-63, -15)
			Cole-Kripke 1	39 (29)	-35 (-58, -13)
			Cole-Kripke 2	8 (8)	-66 (-89, -43)
			Cole-Kripke 3	**55 (56)**	**-19 (-45, 7)**
			Tudor-Locke 1	34 (34)	-40 (-64, -16)
			Tudor-Locke 2	37 (18)	-36 (-60, -13)
			Tudor-Locke 3	29 (24)	-45 (-69, -22)
			Tudor-Locke 4	38 (21)	-36 (-59, -13)
			HDCZA	**58 (47)**	**-16 (-42, 10)**
	PSG	*n* = 119		71 (120)	Reference
	Actigraph GT3x	Wrist (*n* = 119)	Count-scaled	51 (20)	-20 (-42, 2)
			Sadeh 1	**63 (31)**	**-8 (-30, 13)**
			Sadeh 2	**66 (65)**	**-5 (-30, 20)**
			Cole-Kripke 1	130 (50)	58 (36, 81)
			Cole-Kripke 2	42 (25)	-30 (-51, -8)
			Cole-Kripke 3	**51 (55)**	**-20 (-44, 4)**
			Tudor-Locke 1	**50 (53)**	**-22 (-45, 2)**
			Tudor-Locke 2	49 (28)	-22 (-44, -1)
			Tudor-Locke 3	**65 (59)**	**-6 (-31, 18)**
			Tudor-Locke 4	**58 (24)**	**-13 (-34, 8)**
			HDCZA	**81 (60)**	**10 (-14, 35)**
Sleep efficiency_SPT_ (%)	PSG	*n* = 114		95.0 (4.1)	Reference
	Actigraph GT3x	Hip (*n* = 114)	Count-scaled	92.8 (3.1)	-2.2 (-3.1, -1.4)
			Sadeh 1	97.0 (2.8)	2.0 (1.2, 2.7)
			Sadeh 2	**95.1 (3.5)**	**0.0 (-0.8, 0.9)**
			Cole-Kripke 1	93.2 (5.0)	-1.8 (-2.8, -0.8)
			Cole-Kripke 2	98.6 (1.4)	3.6 (2.9, 4.3)
			Cole-Kripke 3	93.9 (4.6)	-1.1 (-2.2, -0.1)
			Tudor-Locke 1	95.9 (2.6)	0.8 (0.0, 1.7)
			Tudor-Locke 2	**94.9 (2.4)**	**-0.1 (-1.0, 0.8)**
			Tudor-Locke 3	**95.8 (2.8)**	**0.7 (-0.1, 1.6)**
			Tudor-Locke 4	**94.4 (3.0)**	**-0.6 (-1.5, 0.3)**
			HDCZA	89.5 (8.6)	-5.5 (-7.2, -3.7)
	PSG	*n* = 119		94.9 (4.1)	Reference
	Actigraph GT3x	Wrist (*n* = 119)	Count-scaled	90.8 (3.3)	-4.2 (-5.0, -3.4)
			Sadeh 1	88.9 (5.1)	-6.0 (-7.0, -5.1)
			Sadeh 2	88.8 (5.9)	-6.1 (-7.2, -5.0)
			Cole-Kripke 1	76.6 (8.3)	-18.4 (-19.9, -16.8)
			Cole-Kripke 2	92.7 (4.0)	-2.2 (-3.1, -1.3)
			Cole-Kripke 3	92.0 (5.1)	-2.9 (-3.9, -1.9)
			Tudor-Locke 1	92.4 (4.8)	-2.5 (-3.5, -1.6)
			Tudor-Locke 2	91.9 (4.1)	-3.0 (-3.8, -2.1)
			Tudor-Locke 3	89.6 (5.4)	-5.4 (-6.3, -4.4)
			Tudor-Locke 4	89.9 (3.9)	-5.1 (-5.9, -4.3)
			HDCZA	85.9 (10.3)	-9.1 (-11.1, -7.1)
Sleep efficiency_TIB_ (%)	PSG	*n* = 114		89.1 (12.5)	Reference
	Actigraph GT3x	Hip (*n* = 114)	Count-scaled	91.6 (9.8)	**2.5 (-0.1, 5.0)**
			Sadeh 1	95.9 (4.6)	6.8 (4.4, 9.2)
			Sadeh 2	95.1 (3.5)	5.9 (3.6, 9.3)
			Cole-Kripke 1	90.7 (6.4)	**1.6 (-0.9, 4.1)**
			Cole-Kripke 2	98.2 (3.0)	9.1 (6.7, 11.4)
			Cole-Kripke 3	93.9 (4.6)	4.8 (2.3, 7.2)
			Tudor-Locke 1	95.9 (2.6)	6.7 (4.3, 9.2)
			Tudor-Locke 2	94.9 (2.4)	5.8 (3.4, 8.2)
			Tudor-Locke 3	95.8 (2.8)	6.6 (4.3, 9.0)
			Tudor-Locke 4	94.4 (3.0)	5.3 (2.9, 7.7)
			HDCZA	NA	NA
	PSG	*n* = 119		89.2 (12.5)	
	Actigraph GT3x	Wrist (*n* = 119)	Count-scaled	86.5 (7.8)	-2.8 (-5.1, -0.4)
			Sadeh 1	85.5 (7.4)	-3.7 (-6.0, -1.5)
			Sadeh 2	88.8 (5.9)	-**0.4 (-2.8, 2.0)**
			Cole-Kripke 1	72.2 (9.3)	-17.0 (-19.7, -14.3)
			Cole-Kripke 2	90.0 (6.6)	**0.8 (-1.3, 2.9)**
			Cole-Kripke 3	92.0 (5.1)	2.8 (0.4, 5.1)
			Tudor-Locke 1	92.4 (4.8)	3.2 (0.8, 5.5)
			Tudor-Locke 2	91.9 (4.1)	2.7 (0.4, 5.1)
			Tudor-Locke 3	89.6 (5.4)	**0.4 (-2.0, 2.7)**
			Tudor-Locke 4	89.9 (3.9)	**0.6 (-1.6, 2.9)**
			HDCZA	NA	NA
Night wakings (frequency)	PSG	*n* = 114		25.8 (8.3)	Reference
	Actigraph GT3x	Hip (*n* = 114)	Count-scaled	0.4 (0.6)	-25.4 (-27.0, -23.9)
			Sadeh 1	0.2 (0.5)	-25.6 (-27.2, -24.1)
			Sadeh 2	10.4 (6.7)	-15.5 (-17.2, -13.7)
			Cole-Kripke 1	13.5 (7.8)	-12.3 (-14.0, -10.5)
			Cole-Kripke 2	0.0 (0.2)	-25.8 (-27.3, -24.2)
			Cole-Kripke 3	13.2 (8.1)	-12.6 (-14.7, -10.6)
			Tudor-Locke 1	10.9 (7.0)	-14.9 (-16.7, -13.1)
			Tudor-Locke 2	11.0 (4.5)	-14.8 (-16.4, -13.2)
			Tudor-Locke 3	11.4 (7.7)	-14.4 (-16.1, -12.6)
			Tudor-Locke 4	12.6 (7.0)	-13.2 (-14.9, -11.4)
			HDCZA	17.1 (5.7)	-8.7 (-10.2, -7.2)
	PSG	*n* = 119		26.3 (8.4)	Reference
	Actigraph GT3x	Wrist (*n* = 119)	Count-scaled	0.7 (0.8)	-25.6 (-27.1, -24.1)
			Sadeh 1	1.0 (1.1)	-25.3 (-26.7, -23.8)
			Sadeh 2	20.4 (7.5)	-5.9 (-7.4, -4.3)
			Cole-Kripke 1	**25.1 (7.2)**	**-1.2 (-2.8, 0.4)**
			Cole-Kripke 2	0.3 (0.6)	-26.0 (-27.5, -24.5)
			Cole-Kripke 3	17.9 (8.5)	-8.4 (-10.1, -6.7)
			Tudor-Locke 1	19.2 (9.3)	-7.1 (-8.9, -5.4)
			Tudor-Locke 2	20.1 (8.6)	-6.2 (-7.8, -4.6)
			Tudor-Locke 3	24.7 (10.0)	-1.6 (-3.5, -0.2)
			Tudor-Locke 4	25.1 (9.0)	-1.2 (-2.8, 0.4)
			HDCZA	20.6 (6.2)	-5.7 (-7.1, -4.2)

*NA* not available

^a^Bolded differences refer to those whether actigraphy was not significantly different (*P* > 0.05) to PSG, and thus provide a good estimate for that sleep measure

**Table 5:** Comparison of PSG and Axivity measured sleep outcomes using different algorithms and at each site

Sleep variable	Device	Placement	Algorithm	Mean (SD)	Mean difference (95% CI) from PSG^a^
Sleep onset (hh:mm)	PSG	(*n* = 116)		21:41 (58)	Reference
	Axivity	Thigh (*n* = 116)	Count-scaled	21:28 (63)	-14 (-22, -5)
			Sadeh 1	21:34 (62)	-8 (-14, -1)
			Sadeh 2	20:57 (102)	-44 (-62, -27)
			Cole-Kripke 1	21:31 (52)	-10 (-15, -5)
			Cole-Kripke 2	21:23 (54)	-18 (-22, -14)
			Cole-Kripke 3	20:17 (115)	-84 (-104, -64)
			Tudor-Locke 1	20:40 (100)	-61 (-78, -45)
			Tudor-Locke 2	20:15 (59)	-86 (-99, -73)
			Tudor-Locke 3	21:07 (100)	-34 (-51, -17)
			Tudor-Locke 4	20:50 (62)	-52 (-62, -42)
			HDCZA	21:09 (101)	-33 (-48, -18)
	PSG	*n* = 115		21:43 (58)	Reference
	Axivity	Back (*n* = 115)	Count-scaled	21:22 (54)	-20 (-28, -13)
			Sadeh 1	21:28 (58)	-15 (-20, -9)
			Sadeh 2	20:29 (104)	-73 (-92, -55)
			Cole-Kripke 1	21:28 (52)	-15 (-19, -10)
			Cole-Kripke 2	21:25 (56)	-18 (-22, -13)
			Cole-Kripke 3	19:19 (142)	-144 (-172, -116)
			Tudor-Locke 1	19:51 (136)	-112 (-137, -86)
			Tudor-Locke 2	19:52 (59)	-111 (-125, -97)
			Tudor-Locke 3	20:45 (102)	-57 (-75, -40)
			Tudor-Locke 4	20:21 (67)	-79 (-91, -67)
			HDCZA	21:26 (101)	-21 (-37, -5)
	PSG	*n* = 125		21:39 (57)	Reference
	Axivity	Wrist (*n* = 125)	Count-scaled	**21:32 (63)**	**-6 (-14, 2)**
			Sadeh 1	**21:40 (65)**	**2 (-6, 10)**
			Sadeh 2	21:13 (96)	-25 (-39, -10)
			Cole-Kripke 1	**21:39 (61)**	**1 (-5, 7)**
			Cole-Kripke 2	21:30 (56)	-9 (-14, -3)
			Cole-Kripke 3	20:56 (94)	-43 (-57, -28)
			Tudor-Locke 1	21:05 (95)	-34 (-48, -20)
			Tudor-Locke 2	20:54 (60)	-44 (-53, -35)
			Tudor-Locke 3	**21:30 (100)**	**-9 (-24, 6)**
			Tudor-Locke 4	21:12 (66)	-27 (-34, -20)
			HDCZA	21:01 (113)	-38 (-54, -21)
Sleep offset (hh:mm)	PSG	*n* = 116		6:40 (92)	Reference
	Axivity	Thigh (*n* = 116)	Count-scaled	**6:42 (65)**	**2 (-12, 17)**
			Sadeh 1	**6:44 (59)**	**5 (-9, 18)**
			Sadeh 2	**6:58 (98)**	**18 (-4, 40)**
			Cole-Kripke 1	**6:40 (65)**	**0 (-14, 14)**
			Cole-Kripke 2	**6:49 (57)**	**9 (-4, 23)**
			Cole-Kripke 3	7:26 (98)	47 (23, 70)
			Tudor-Locke 1	7:14 (99)	35 (12, 57)
			Tudor-Locke 2	7:12 (87)	32 (11, 53)
			Tudor-Locke 3	**6:54 (98)**	**14 (-8, 36)**
			Tudor-Locke 4	**6:45 (73)**	**6 (-13, 24)**
			HDCZA	**6:35 (120)**	**-4 (-29, 21)**
	PSG	*n* = 115		6:42 (95)	Reference
	Axivity	Back (*n* = 115)	Count-scaled	**6:47 (69)**	**5 (-10, 20)**
			Sadeh 1	**6:49 (61)**	**7 (-7, 21)**
			Sadeh 2	7:34 (107)	52 (28, 76)
			Cole-Kripke 1	**6:49 (62)**	**8 (-6, 22)**
			Cole-Kripke 2	6:55 (61)	14 (0, 27)
			Cole-Kripke 3	8:01 (128)	79 (51, 106)
			Tudor-Locke 1	7:57 (106)	75 (51, 99)
			Tudor-Locke 2	7:41 (93)	59 (38, 80)
			Tudor-Locke 3	7:22 (107)	41 (17, 64)
			Tudor-Locke 4	7:11 (88)	29 (9, 49)
			HDCZA	**6:34 (108)**	**-8 (-32, 17)**
	PSG	*n* = 125		6:40 (92)	Reference
	Axivity	Wrist (*n* = 125)	Count-scaled	**6:35 (70)**	**-5 (-19, 10)**
			Sadeh 1	**6:39 (62)**	**-1 (-15, 12)**
			Sadeh 2	**6:22 (103)**	**-18 (-39, 4)**
			Cole-Kripke 1	**6:31 (73)**	**-9 (-23, 6)**
			Cole-Kripke 2	**6:41 (61)**	**1 (-12, 15)**
			Cole-Kripke 3	**6:47 (97)**	**7 (-14, 28)**
			Tudor-Locke 1	**6:40 (96)**	**0 (-21, 21)**
			Tudor-Locke 2	**6:31 (71)**	**-9 (-24, 7)**
			Tudor-Locke 3	6:07 (116)	-33 (-57, -9)
			Tudor-Locke 4	6:19 (73)	-21 (-39, -4)
			HDCZA	**6:21 (143)**	**-19 (-47, 8)**
Sleep period time (min)	PSG	*n* = 116		538 (95)	Reference
	Axivity	Thigh (*n* = 116)	Count-scaled	555 (78)	16 (-1, 33)
			Sadeh 1	**550 (69)**	**12 (-2, 26)**
			Sadeh 2	602 (130)	64 (34, 93)
			Cole-Kripke 1	**549 (69)**	**10 (-4, 25)**
			Cole-Kripke 2	566 (63)	27 (13, 41)
			Cole-Kripke 3	670 (139)	132 (100, 163)
			Tudor-Locke 1	657 (115)	97 (70, 124)
			Tudor-Locke 2	658 (107)	119 (95, 144)
			Tudor-Locke 3	588 (128)	49 (20, 78)
			Tudor-Locke 4	597 (88)	58 (37, 79)
			HDCZA	567 (86)	28 (8, 49)
	PSG	*n* = 115		539 (97)	Reference
	Axivity	Back (*n* = 115)	Count-scaled	564 (77)	25 (10, 41)
			Sadeh 1	561 (68)	22 (7, 37)
			Sadeh 2	665 (153)	126 (93, 160)
			Cole-Kripke 1	562 (67)	23 (8, 37)
			Cole-Kripke 2	570 (63)	31 (18, 45)
			Cole-Kripke 3	763 (187)	224 (185, 263)
			Tudor-Locke 1	727 (172)	188 (150, 225)
			Tudor-Locke 2	710 (117)	171 (146, 196)
			Tudor-Locke 3	638 (146)	99 (67, 131)
			Tudor-Locke 4	648 (109)	109 (87, 132)
			HDCZA	**553 (90)**	**14 (-8, 35)**
	PSG	*n* = 125		541 (95)	Reference
	Axivity	Wrist (*n* = 125)	Count-scaled	**543 (81)**	**1 (-13, 16)**
			Sadeh 1	**538 (78)**	**-3 (-18, 12)**
			Sadeh 2	**549 (113)**	**8 (-16, 32)**
			Cole-Kripke 1	**532 (72)**	**-10 (-24, 5)**
			Cole-Kripke 2	**551 (71)**	**10 (-3, 23)**
			Cole-Kripke 3	592 (109)	51 (27, 74)
			Tudor-Locke 1	576 (104)	35 (12, 58)
			Tudor-Locke 2	578 (74)	37 (20, 53)
			Tudor-Locke 3	**519 (122)**	**-23 (-49, 4)**
			Tudor-Locke 4	**548 (72)**	**7 (-10, 24)**
			HDCZA	**560 (95)**	**18 (-2, 39)**
Total sleep time (mins)	PSG	*n* = 116		511 (93)	Reference
	Axivity	Thigh (*n* = 116)	Count-scaled	**507 (76)**	**-4 (-21, 13)**
			Sadeh 1	**518 (72)**	**7 (-9, 23)**
			Sadeh 2	564 (105)	53 (28, 78)
			Cole-Kripke 1	480 (71)	-31 (-48, -14)
			Cole-Kripke 2	545 (65)	36 (21, 51)
			Cole-Kripke 3	633 (116)	122 (95, 149)
			Tudor-Locke 1	610 (103)	99 (75, 124)
			Tudor-Locke 2	619 (100)	109 (86, 132)
			Tudor-Locke 3	554 (104)	43 (18, 68)
			Tudor-Locke 4	556 (83)	45 (25, 66)
			HDCZA	**498 (72)**	**-12 (-30, 6)**
	PSG	*n* = 115		511 (94)	Reference
	Axivity	Back (*n* = 115)	Count-scaled	533 (76)	22 (6, 37)
			Sadeh 1	546 (69)	35 (20, 50)
			Sadeh 2	634 (129)	123 (94, 151)
			Cole-Kripke 1	529 (68)	18 (2, 33)
			Cole-Kripke 2	560 (64)	49 (35, 63)
			Cole-Kripke 3	716 (160)	205 (171, 239)
			Tudor-Locke 1	693 (149)	182 (149, 215)
			Tudor-Locke 2	675 (112)	164 (140, 189)
			Tudor-Locke 3	614 (126)	103 (75, 132)
			Tudor-Locke 4	618 (101)	107 (85, 129)
			HDCZA	498 (80)	**-13 (-31, 6)**
	PSG	*n* = 125		514 (92)	Reference
	Axivity	Wrist (*n* = 125)	Count-scaled	491 (80)	-22 (-38, -6)
			Sadeh 1	472 (77)	-41 (-58, -25)
			Sadeh 2	**491 (100)**	**-22 (-44, 0)**
			Cole-Kripke 1	404 (66)	-110 (-127, -92)
			Cole-Kripke 2	**506 (73)**	**-8 (-23, 7)**
			Cole-Kripke 3	547 (97)	33 (11, 55)
			Tudor-Locke 1	535 (93)	21 (0, 43)
			Tudor-Locke 2	531 (72)	17 (1, 33)
			Tudor-Locke 3	691 (111)	-45 (-70, -20)
			Tudor-Locke 4	489 (70)	-24 (-41, -7)
			HDCZA	492 (78)	-22 (-39, -5)
WASO (min)	PSG	*n* = 116		74 (125)	Reference
	Axivity	Thigh (*n* = 116)	Count-scaled	48 (22)	-26 (-49, -3)
			Sadeh 1	33 (29)	-41 (-65, -18)
			Sadeh 2	38 (40)	-36 (-59, -13)
			Cole-Kripke 1	**69 (46)**	**-6 (-29, 18)**
			Cole-Kripke 2	19 (22)	-55 (-78, -32)
			Cole-Kripke 3	37 (37)	-37 (-60, -13)
			Tudor-Locke 1	26 (22)	-49 (-71, -26)
			Tudor-Locke 2	38 (21)	-36 (-59, -13)
			Tudor-Locke 3	34 (40)	-40 (-64, -17)
			Tudor-Locke 4	41 (25)	-33 (-56, -11)
			HDCZA	**69 (37)**	**-5 (-29, 18)**
	PSG	*n* = 115		76 (126)	Reference
	Axivity	Back (*n* = 115)	Count-scaled	32 (16)	-45 (-68, -21)
			Sadeh 1	15 (18)	-62 (-84, -39)
			Sadeh 2	32 (41)	-44 (-69, -20)
			Cole-Kripke 1	33 (32)	-44 (-67, -21)
			Cole-Kripke 2	10 (13)	-66 (-90, -43)
			Cole-Kripke 3	47 (48)	-30 (-55, -4)
			Tudor-Locke 1	33 (38)	-43 (-68, -18)
			Tudor-Locke 2	35 (20)	-42 (-65, -18)
			Tudor-Locke 3	24 (31)	-31 (-76, -30)
			Tudor-Locke 4	30 (20)	-46 (-70, -23)
			HDCZA	**54 (27)**	**-22 (-46, 2)**
	PSG	*n* = 125		71 (119)	Reference
	Axivity	Wrist (*n* = 125)	Count-scaled	**51 (28)**	**-19 (-41, 2)**
			Sadeh 1	**66 (43)**	**-5 (-26, 17)**
			Sadeh 2	**58 (29)**	**-13 (-34, 9)**
			Cole-Kripke 1	128 (48)	57 (36, 79)
			Cole-Kripke 2	46 (39)	-25 (-46, -3)
			Cole-Kripke 3	45 (26)	-25 (-46, -4)
			Tudor-Locke 1	42 (26)	-29 (-50, -8)
			Tudor-Locke 2	47 (24)	-23 (-44, -3)
			Tudor-Locke 3	**50 (24)**	**-20 (-42, 1)**
			Tudor-Locke 4	**59 (25)**	**-12 (-32, 8)**
			HDCZA	**68 (39)**	**-3 (-24, 19)**
Sleep efficiency_SPT_ (%)	PSG	*n* = 116		94.8 (4.3)	Reference
	Axivity	Thigh (*n* = 116)	Count-scaled	91.3 (3.8)	-3.5 (-4.4, -2.6)
			Sadeh 1	**94.0 (5.6)**	**-0.8 (-2.0, 0.5)**
			Sadeh 2	**94.0 (4.1)**	**-0.8 (-1.8, 0.2)**
			Cole-Kripke 1	87.6 (7.8)	-7.2 (-8.7, -5.7)
			Cole-Kripke 2	96.7 (3.9)	1.9 (0.9, 2.9)
			Cole-Kripke 3	**94.8 (4.0)**	**0.0 (-1.0, 1.0)**
			Tudor-Locke 1	96.2 (2.7)	1.4 (0.5, 2.3)
			Tudor-Locke 2	**94.3 (3.1)**	**-0.6 (-1.4, 0.3)**
			Tudor-Locke 3	**94.6 (4.1)**	**-0.2 (-1.2, 0.7)**
			Tudor-Locke 4	93.2 (4.1)	-1.6 (-2.5, -0.6)
			HDCZA	88.2 (5.1)	-6.6 (-7.7, -5.6)
	PSG	*n* = 115		94.8 (4.3)	Reference
	Axivity	Back (*n* = 115)	Count-scaled	**94.3 (2.9)**	**-0.5 (-1.3, 0.4)**
			Sadeh 1	97.3 (3.2)	2.6 (1.7, 3.4)
			Sadeh 2	**95.6 (4.2)**	**0.9 (-0.1, 1.8)**
			Cole-Kripke 1	**94.3 (5.5)**	**-0.5 (-1.7, 0.6)**
			Cole-Kripke 2	98.2 (2.2)	3.5 (2.6, 4.3)
			Cole-Kripke 3	**94.4 (4.6)**	**-0.4 (-1.4, 0.6)**
			Tudor-Locke 1	95.8 (3.7)	1.1 (0.1, 2.0)
			Tudor-Locke 2	**95.1 (2.7)**	**0.3 (-0.5, 1.2)**
			Tudor-Locke 3	96.6 (3.1)	1.9 (1.0, 2.7)
			Tudor-Locke 4	**95.5 (2.9)**	**0.7 (-0.2, 1.5)**
			HDCZA	90.3 (4.3)	-4.5 (-5.5, -3.5)
	PSG	*n* = 125		94.8 (4.2)	Reference
	Axivity	Wrist (*n* = 125)	Count-scaled	90.5 (5.5)	-4.3 (-5.4, -3.2)
			Sadeh 1	87.8 (8.0)	-7.0 (-8.5, -5.5)
			Sadeh 2	89.5 (4.3)	-5.3 (-6.2, -4.4)
			Cole-Kripke 1	76.1 (8.3)	-18.8 (-20.3, -17.2)
			Cole-Kripke 2	91.8 (7.3)	-3.1 (-4.5, -1.6)
			Cole-Kripke 3	92.5 (3.6)	-2.4 (-3.2, -1.5)
			Tudor-Locke 1	92.9 (3.6)	-1.9 (-2.7, -1.0)
			Tudor-Locke 2	91.8 (4.0)	-3.0 (-3.9, -2.1)
			Tudor-Locke 3	90.3 (3.9)	-4.5 (-5.3, -3.7)
			Tudor-Locke 4	89.3 (4.5)	-5.6 (-6.5, -4.6)
			HDCZA	88.1 (5.2)	-6.7 (-7.7, -5.7)
Sleep efficiency_TIB_ (%)	PSG	*n* = 116		88.8 (9.6)	
	Axivity	Thigh (*n* = 116)	Count-scaled	88.2 (9.6)	**-0.5 (-3.4, 2.3)**
			Sadeh 1	90.1 (8.7)	**1.4 (-1.3, 4.0)**
			Sadeh 2	94.0 (4.1)	5.3 (2.9, 7.6)
			Cole-Kripke 1	83.6 (9.3)	-5.2 (-8.0, -2.4)
			Cole-Kripke 2	95.1 (5.2)	6.4 (3.9, 8.9)
			Cole-Kripke 3	94.8 (4.0)	6.1 (3.7, 8.4)
			Tudor-Locke 1	96.2 (2.7)	7.4 (5.1, 9.8)
			Tudor-Locke 2	94.3 (3.1)	5.5 (3.1, 7.9)
			Tudor-Locke 3	94.6 (4.1)	5.8 (3.5, 8.2)
			Tudor-Locke 4	93.2 (4.1)	4.5 (2.1, 6.8)
			HDCZA	NA	NA
	PSG	*n* = 115		88.8 (12.7)	
	Axivity	Back (*n* = 115)	Count-scaled	92.7 (9.1)	3.9 (1.3, 6.5)
			Sadeh 1	95.0 (6.5)	6.2 (3.5, 8.8)
			Sadeh 2	95.6 (4.2)	6.9 (4.5, 9.2)
			Cole-Kripke 1	92.0 (6.5)	3.2 (0.6, 5.8)
			Cole-Kripke 2	97.5 (4.2)	8.7 (6.3, 11.1)
			Cole-Kripke 3	94.3 (4.6)	5.6 (3.1, 8.1)
			Tudor-Locke 1	95.8 (3.7)	7.1 (4.7, 9.4)
			Tudor-Locke 2	95.1 (2.7)	6.3 (3.9, 8.8)
			Tudor-Locke 3	96.6 (3.1)	7.9 (5.5, 10.2)
			Tudor-Locke 4	95.5 (2.9)	6.7 (4.3, 9.1)
			HDCZA	NA	NA
	PSG	*n* = 125		88.8 (12.4)	
	Axivity	Wrist (*n* = 125)	Count-scaled	85.0 (10.2)	-3.9 (-6.5, -1.2)
			Sadeh 1	81.7 (10.5)	-7.1 (-9.9, -4.3)
			Sadeh 2	89.5 (4.3)	**0.7 (-1.6, 2.9)**
			Cole-Kripke 1	70.1 (10.4)	-18.7 (-21.5, -15.9)
			Cole-Kripke 2	87.5 (9.1)	**-1.3 (-3.9, 1.3)**
			Cole-Kripke 3	92.5 (3.6)	3.6 (1.4, 5.9)
			Tudor-Locke 1	92.9 (3.6)	4.1 (1.9, 6.3)
			Tudor-Locke 2	91.8 (4.0)	3.0 (0.8, 5.2)
			Tudor-Locke 3	90.3 (3.9)	**1.5 (-0.7, 3.7)**
			Tudor-Locke 4	89.3 (4.5)	**0.4 (-1.8, 2.7)**
			HDCZA	NA	NA
Night wakings (frequency)	PSG	*n* = 116		26.0 (8.3)	Reference
	Axivity	Thigh (*n* = 116)	Count-scaled	0.6 (0.9)	-25.4 (-26.9, -23.9)
			Sadeh 1	0.3 (0.6)	-25.7 (-27.2, -24.2)
			Sadeh 2	14.0 (7.9)	-12.0 (-13.9, -10.1)
			Cole-Kripke 1	20.0 (8.1)	-6.0 (-7.8, -4.2)
			Cole-Kripke 2	0.2 (0.5)	-25.9 (-27.4, -24.4)
			Cole-Kripke 3	11.1 (7.6)	-15.0 (-16.9, -13.0)
			Tudor-Locke 1	9.3 (6.4)	-16.7 (-18.5, -14.9)
			Tudor-Locke 2	10.9 (6.1)	-15.1 (-16.8, -13.4)
			Tudor-Locke 3	13.3 (7.8)	-12.7 (-14.6, -10.8)
			Tudor-Locke 4	14.3 (7.4)	-11.7 (-13.4, -10.0)
			HDCZA	19.1 (4.9)	-6.9 (-8.4, -5.5)
	PSG	*n* = 115		26.1 (8.4)	Reference
	Axivity	Back (*n* = 115)	Count-scaled	0.2 (0.5)	-25.9 (-27.4, -24.3)
			Sadeh 1	0.1 (0.3)	**-26.0 (-27.6, 24.5)**
			Sadeh 2	8.4 (5.6)	-17.7 (-19.3, -16.1)
			Cole-Kripke 1	10.6 (6.5)	-15.5 (-17.1, -14.0)
			Cole-Kripke 2	0.1 (0.3)	-26.1 (-27.6, -24.5)
			Cole-Kripke 3	10.6 (6.5)	-15.5 (-17.6, -13.4)
			Tudor-Locke 1	8.8 (5.7)	-17.3 (-19.3, -15.3)
			Tudor-Locke 2	8.8 (4.2)	-17.3 (-19.0, -15.6)
			Tudor-Locke 3	7.4 (5.2)	-18.7 (-20.4, -17.1)
			Tudor-Locke 4	8.4 (5.0)	-17.7 (-19.3, -16.2)
			HDCZA	17.5 (4.8)	-8.7 (-10.2, -7.2)
	PSG	*n* = 125		26.0 (8.1)	Reference
	Axivity	Wrist (*n* = 125)	Count-scaled	0.6 (0.8)	-25.4 (-26.8, -24.0)
			Sadeh 1	0.9 (0.9)	-25.1 (-26.5, -23.7)
			Sadeh 2	20.4 (6.9)	-5.6 (-7.1, -4.1)
			Cole-Kripke 1	24.8 (7.2)	**-1.2 (-2.8, 0.5)**
			Cole-Kripke 2	0.3 (0.6)	-25.7 (-27.1, -24.3)
			Cole-Kripke 3	17.7 (7.1)	-8.3 (-9.8, -6.8)
			Tudor-Locke 1	17.0 (7.2)	-9.0 (-10.5, -7.5)
			Tudor-Locke 2	18.1 (7.4)	-7.9 (-9.4, -6.4)
			Tudor-Locke 3	18.7 (6.5)	-7.3 (-8.8, -5.7)
			Tudor-Locke 4	20.8 (6.9)	-5.3 (-6.7, -3.8)
			HDCZA	19.8 (5.5)	-6.2 (-7.6, -4.9)

^a^Bolded differences refer to those whether actigraphy was not significantly different (*P* > 0.05) to PSG, and thus provide a good estimate for that sleep measure

### Sleep timing

For sleep onset, almost all algorithms detected a sleep onset significantly earlier than the PSG gold standard, with differences ranging from just 2 min to as much as 149 min for the Actigraph and 1 min to 144 min for the Axivity. Overall, differences in sleep onset were generally smaller for either device when placed at the wrist, with several algorithms providing valid estimates of sleep onset with differences of just 1–15 min compared to PSG (Actigraph hip HDCZA, Actigraph wrist CS, Sadeh 1, Sadeh 2, Tudor-Locke 3, Axivity wrist CS, Sadeh 1, Cole-Krikpe 1, Tudor-Locke 3). In terms of sleep offset, differences were smaller for Actigraphs placed at the wrist than those at the hip, with all algorithms except for Tudor-Locke 2 showing small differences compared with PSG. In general, differences for the Axivity placed at the wrist were smaller than those placed at the thigh or back. However, overall, it can be seen that the Axivity placed on the thigh and to a lesser extent on the back, perform better than Actigraph at the hip, with 8 and 4 of 11 algorithms respectively reporting only small, non-significant differences compared to PSG, whereas just one algorithm (HDCZA) produced small differences with the Actigraph placed at the hip.

### Sleep quantity

Tables 4 and 5 demonstrate that many of the algorithms show large differences compared with PSG, in some cases overestimating sleep by more than two hours whether measured as sleep period time or total sleep time. However, there was a clear pattern of wrist placement providing substantially more accurate estimates of sleep quantity, for both devices. For example, differences (95% CI) for the Actigraph at the wrist ranged from 1 (-12, 15) to 54 (27, 81) minutes for sleep period time, whereas the corresponding values for hip placement were up to 243 (203, 283) minutes different. A similar pattern is shown for the Axivity (Table 5). While several algorithms performed well only a few (Sadeh 1, Cole-Kripke 1, HDCZA), consistently performed well for both devices and placement sites and only the count-scaled algorithm showed a difference with PSG of less than 30 min for all eight measures examined (total sleep time and sleep period time at both wrist and hip for both devices).

### Sleep quality

In terms of WASO, examination of Tables 4 and 5 demonstrate that actigraphy produces lower values for WASO compared with PSG for almost all sites, devices and algorithms tested. However, in general, estimates more closely matched PSG values when the device was placed at the wrist, particularly for the Actigraph, with 7 of the 11 algorithms showing small differences (differences ranging from just 5 to 22 min for these algorithms). On the other hand, better estimates of sleep efficiency were obtained from devices placed on the hip (Actigraph), thigh or back (Axivity). Regardless of device or placement, the algorithms tested resulted in small differences in sleep efficiency compared to PSG. Overall, sleep efficiency defined using TIB was lower than sleep efficiency defined using SPT and resulted in larger differences compared to PSG. Lastly, estimates of the number of night wakings differed considerably from PSG measures for most of the algorithms examined. Only 1 of the 20 Actigraph (Cole-Kripke 1 at the wrist) and 2 of the 30 Axivity (Sadeh 1 at the back and Cole-Kripke 1 at the wrist) algorithms tested did not produce large differences in waking frequency (Tables 4 and 5).

### Bland–Altman

**Fig. 1:**
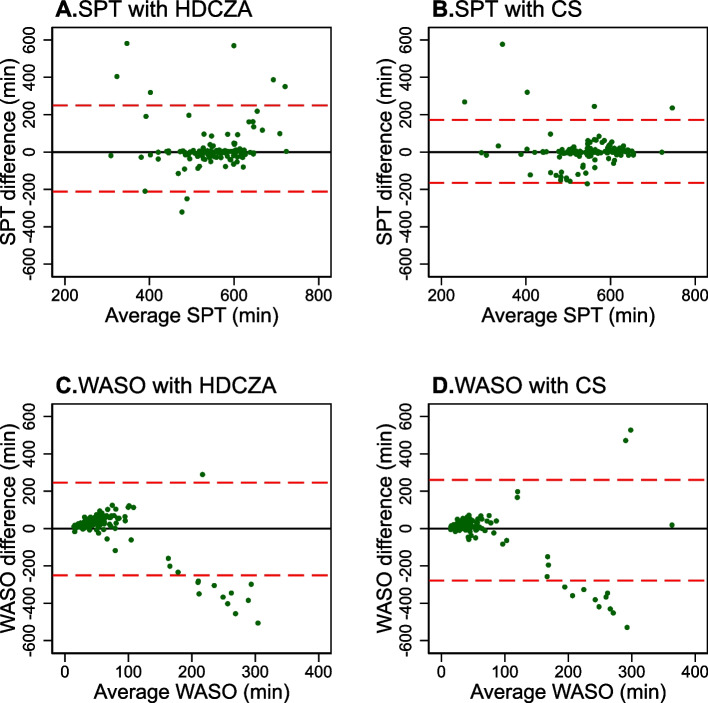
Bland–Altman plots for sleep period time (SPT) and wake after sleep onset (WASO) for the HDCZA and CS algorithms using the Axivity at the wrist compared to PSG. Red dashed lines indicate 95% limits of agreement

Figure 1 shows the Bland–Altman plots for agreement in SPT (a metric for sleep duration) and WASO (a metric for sleep quality) for the ‘overall best performing algorithm’ (HDCZA with the Axivity at the wrist), and the CS algorithm (which was the ‘best performing’ for the Axivity at the wrist for SPT). These plots illustrate that the CS algorithm performs better than the HDCZA for accurate assessment of SPT, with narrower 95% limits of agreement (LOA) (-165 to 172 min compared to -212 to 250 min for HDCZA). Both algorithms demonstrated similar performance for assessing WASO, with slightly lower 95% LOA for HDCZA (CS: -279 to 260 min; and HDCZA: -251 to 245 min) but both showed considerable inaccuracy in determining WASO at higher levels.

## Discussion

Our study demonstrates that current count-based sleep algorithms show higher total accuracy and specificity when devices were placed at the wrist compared with other sites of wear, regardless of actigraphy brand or algorithm tested. Overall, the HDCZA algorithm demonstrated high levels of sensitivity, specificity and thus accuracy regardless of device brand or placement. In terms of the range of sleep outcomes studied, results were more variable and differed across outcomes of interest, algorithm and site of wear. Thus, researchers may choose a certain algorithm over another depending on their primary sleep outcome of interest; for example, studies of sleep timing may prefer the CS algorithm placed at the wrist, whereas studies more focussed on sleep quality may prefer the HDCZA algorithm. Poorer detection of wakefulness (poor specificity) by many of the algorithms and sites of wear continues to plague actigraphy estimates of both sleep and wake in paediatric studies [21] but specificity values are not always reported [22] despite the potential to influence data interpretation. This is also an issue in the adult field [23].

Several studies have assessed the agreement between research grade devices and PSG in healthy children, but many have been in small samples and utilised single sites of wear, devices or algorithms to detect sleep and wake states and derive sleep estimates [10, 20, 22]. Most of the sleep detection algorithms used in the present study have been previously developed and validated against PSG in healthy adults [5, 9, 13], and only a few have been validated against PSG in children [7, 10] albeit in small samples (*n* < 40). The findings from this much larger and more comprehensive study are broadly consistent with the original validation studies and a review of previous validation studies in children, which show that accuracy (0.84–0.92) and sensitivity (0.82–0.96) are generally good, whereas specificity (0.20–0.65) is considerably lower [24].

However it is clear from both previous research and the current study that the specificity (54–77%) [24] or ability to detect periods of wakefulness in the sleep period window, of most algorithms was better when the device was worn at the wrist, with estimates ranging from 67 to 90%. These figures are considerably higher than those observed in adult studies, which have reported specificities of 34–46% for the HDCZA, Sadeh and Cole algorithms when validated in adult samples [9, 11, 25]. These discrepancies may arise because of differences in sleep characteristics between children and adults. In our study, most children had long periods of sleep without wakefulness during the night. Although immobility generally infers sleep in accelerometery-based assessment, immobility is possible during periods of wakefulness and as such can be mistakenly identified as sleep by actigraphy; it is likely this occurs more in adults because they have more periods of conscious nocturnal awakenings than children [11, 26]. Our Bland–Altman plots also revealed some bias between actigraphy-measured sleep period time and PSG, where larger differences were apparent as sleep period time decreased. More wakefulness and the shorter sleep times of adults likely contributes to the greater misclassification of WASO and thus poorer specificity overall compared with children.

The wrist placement was also superior to the thigh, lower back and hip for estimates of sleep onset, offset, quantity (TST and SPT) and WASO for most algorithms. Prior research has also indicated that hip-worn accelerometers tend to overestimate total sleep time and sleep efficiency while underestimating wake after sleep onset (WASO), resulting in lower specificity compared to wrist-worn devices [25, 27]. This reduced specificity for hip-worn devices can be attributed to the algorithms predominantly designed for wrist-specific acceleration features, which are more attuned to nocturnal movements indicative of wakefulness. Devices positioned closer to the body’s center of mass, such as the waist or lower back, are likely to register less movement during the night, potentially leading to overlooked periods of wakefulness. Differing feature selection (y-axis acceleration, inclinometer data, rolling-window size, changes in z-angle, etc.) may also explain why different algorithms outperformed others when devices were worn at the same site. Although we previously reported better estimates of sleep onset using the count-scaled algorithm when devices were worn at the hip [10], this was a much smaller study in younger children, and the very small differences observed (-3 min versus 2 min) may reflect device specific differences or alternatively age-related differences in sleep settling habits. Only sleep efficiency (both definitions) was consistently superior when devices were worn at the hip. Because most algorithms overestimated sleep offset when worn at the hip (i.e. result in later waking), and underestimated WASO, sleep efficiency was thus higher. When determining the most optimal placement, device and algorithm to use, systematic variation should be an important aspect to consider. Systematic variation is more tractable than random variation because the direction of bias is known. In this study, the HDCZA, Sadeh 1, CS, and Cole-Kripke 1 algorithms performed well for estimates of sleep onset, offset, total sleep time and sleep period time, and importantly these estimates did not randomly vary when different devices or placements were used. Knowing that an algorithm, regardless of site placement or device type, always identifies sleep onset before PSG means that actigraphy identifies earlier sleep onset and thus overestimates total sleep time, and in turn, sleep efficiency.

Many current algorithms are disadvantaged by requiring sleep onset and offset times from diaries, which pose both respondent and analysis burden. Therefore, we specifically compared sleep estimates from three different algorithms (Sadeh 1, Cole-Kripke 2, Tudor-Locke 1) with PSG using diary recorded sleep onset and offset timings to guide the algorithm. Overall, the use of a sleep diary did not improve the level of agreement of sleep estimates between accelerometers and PSG. Although the children were asked about their sleep onset and waking times not long after awakening, it appears that estimating these timings by self-report is challenging, particularly estimating timing of sleep onset, and especially when more than one day of data are collected. These findings lend further support for using automated algorithms for detecting sleep and wake states, especially in large sample sizes.

Limitations of our study include that the accuracy in clinical populations or in children with any significant sleep disturbance is unknown, and it is not known whether these results would be similar in other age groups or those with irregular sleep patterns. Although we did not include a direct measure of sleep latency (an important sleep metric), “in-bed” time remained the same across site placement, device and algorithm, which suggests later sleep onsets would result in longer sleep latency.

The strengths of this study include the simultaneous comparison of two research-grade accelerometers worn at several sites (wrist, hip, thigh, lower back) with PSG, the rigorous reporting of actigraphy data according to recommendations for children [22], and the larger number of children included in this validation study than most previous studies [22]. Importantly, sleep data were generated using 11 different automated sleep detection algorithms commonly reported in the literature, but not previously compared to PSG in a large sample of children and adolescents. While the comparison of accelerometers to the “gold-standard” PSG is a strength, it must be acknowledged that these two techniques do measure very different signals and actigraphy sleep scoring rules, particularly for WASO, are not entirely comparable to PSG. This likely explains the discrepancies, alongside the fact that actigraphy can wrongly infer sleep when children are lying awake and relatively motionless. This is particularly relevant as children settle to sleep but are still awake, and likely explains the earlier sleep onset detected by actigraphy. PSG detects sleep using changes in brain wave signals which can occur within a 30 s epoch. This rapid change may also explain the high frequencies of wakings detected by PSG, but not by actigraphy.

The differences between PSG and actigraphy methodology may also explain the large discrepancies between algorithms for estimates such as WASO and number of awakenings. Many of the algorithms define WASO as any transition between sleep and wake after sleep onset and before sleep offset, similar to PSG scoring. However, the CS algorithm aims to minimise artefactual movements detected during sleep by actigraphy and defines WASO as movements that occur over 5 continuous minutes of awake. This method of defining WASO means disagreements between PSG and actigraphy are considerably greater, but it is not clear if estimates of sleep used to demonstrate relationships with various aspects of health are affected by differences in how WASO is defined. To our knowledge, this has not been examined in the literature. Researchers may need to consider whether using a different gold standard measure of sleep, such as videosomnography, that measures similar constructs of sleep as actigraphy in future validation studies. Accurately discriminating between “awake” time and movement during sleep is important if the true relationships between sleep and health are of interest. Future studies where relationships between sleep estimates derived using different sleep algorithms and health should also be evaluated. Likewise, understanding what brand of accelerometer and site placement is best for accurate assessment of sleep may not necessarily align with the best choice for assessing other movement behaviours in the day (such as physical activity and sedentary behaviour). Researchers investigating 24 h movement behaviours will have to consider these results in the context of their objectives.

## Conclusion

In conclusion, our study suggests that automated sleep detection algorithms applied to Actigraph and Axivity accelerometers, worn either at the lower back, hip or thigh, provide moderately comparable measures with PSG, but estimates of sleep outcomes including sleep quantity, sleep onset, sleep offset and WASO improve markedly when accelerometers are worn at the wrist. Accelerometry should be used cautiously in studies where estimates of sleep quality such as sleep efficiency and number of awakenings during sleep period are important or in samples of participants who experience frequent periods of wake after sleep onset.

### Supplementary Information

**Additional file 1: Supplementary Table 1. **Number of participants with missing data from each algorithm (*n*=131). **Supplementary Table 2.** Sensitivity analysis for sensitivity, specificity, and accuracy of epoch-by-epoch comparisons with PSG for sleep with half PSG epochs assigned as sleep (rather than wake).

## Data Availability

Data used in the current study are available and may be obtained from the corresponding author upon reasonable request.

## Abbreviations

PSG: Polysomnography
SDSC: Sleep Disturbances Scale for Children
BMI: Body mass index
EOG: Electro-oculograms
EEG: Electroencephalograms
AASM: American Academy of Sleep Medicine
TST: Total sleep time
SPT: Sleep period time
WASO: Wake after sleep onset
SE: Sleep efficiency
LOA: Limits of agreement
CS: Count-Scaled

## References

[1] Matricciani L, Paquet C, Galland B, Short M, Olds T (2019). Children’s sleep and health: a meta-review. Sleep Med Rev.

[2] Acebo C, Sadeh A, Seifer R, Tzischinsky O, Wolfson AR, Hafer A (1999). Estimating sleep patterns with activity monitoring in children and adolescents: how many nights are necessary for reliable measures?. Sleep..

[3] Lauderdale DS, Knutson KL, Yan LL, Liu K, Rathouz PJ (2008). Self-reported and measured sleep duration: how similar are they?. Epidemiology..

[4] Barreira TV, Schuna JM, Jr., Mire EF, Katzmarzyk PT, Chaput JP, Leduc G, et al. Identifying children's nocturnal sleep using 24-h waist accelerometry. Med Sci Sports Exerc. 2015;47(5):937–43. https://doi.org/910.1249/MSS.0000000000000486. 10.1249/MSS.0000000000000486 25202840

[5] Cole RJ, Kripke DF, Gruen W, Mullaney DJ, Gillin JC (1992). Automatic sleep/wake identification from wrist activity. Sleep..

[6] Galland BC, Kennedy GJ, Mitchell EA, Taylor BJ (2012). Algorithms for using an activity-based accelerometer for identification of infant sleep-wake states during nap studies. Sleep medicine..

[7] Sadeh A, Lavie P, Scher A, Tirosh E, Epstein R (1991). Actigraphic home-monitoring sleep-disturbed and control infants and young children: a new method for pediatric assessment of sleep-wake patterns. Pediatrics..

[8] Tudor-Locke C, Barreira TV, Schuna JM, Jr., Mire EF, Katzmarzyk PT. Fully automated waist-worn accelerometer algorithm for detecting children's sleep-period time separate from 24-h physical activity or sedentary behaviors. Appl Physiol Nutr Metab. 2014;39(1):53–7. 10.1139/apnm-2013-0173. Epub 2013 Jun 1126. 10.1139/apnm-2013-0173 24383507

[9] van Hees VT, Sabia S, Jones SE, Wood AR, Anderson KN, Kivimäki M, et al. Estimating sleep parameters using an accelerometer without sleep diary. Sci Rep. 2018;8(1):12975. 10.1038/s41598-018-31266-z PMC6113241 30154500

[10] Smith C, Galland B, Taylor R, Meredith-Jones K. ActiGraph GT3X+ and Actical Wrist and Hip Worn Accelerometers for Sleep and Wake Indices in Young Children Using an Automated Algorithm: Validation With Polysomnography. Front Psychiatry. 2019;10:958. 10.3389/fpsyt.2019.00958 PMC6970953 31992999

[11] Quante M, Kaplan ER, Cailler M, Rueschman M, Wang R, Weng J, et al. Actigraphy-based sleep estimation in adolescents and adults: a comparison with polysomnography using two scoring algorithms. Nat Sci Sleep. 2018;10:13–20. 10.2147/NSS.S151085. eCollection 152018. 10.2147/NSS.S151085 PMC5779275 29403321

[12] Girschik J, Fritschi L, Heyworth J, Waters F. Validation of Self-Reported Sleep Against Actigraphy. J Epidemiol. 2012;22(5):462–8. 10.2188/jea.JE20120012 PMC3798642 22850546

[13] Sadeh A, Sharkey KM, Carskadon MA. Activity-based sleep-wake identification: an empirical test of methodological issues. Sleep. 1994;17(3):201–7. https://doi.org/210.1093/sleep/1017.1093.1201. 10.1093/sleep/17.3.201 7939118

[14] Bruni O, Ottaviano S, Guidetti V, Romoli M, Innocenzi M, Cortesi F, et al. The Sleep Disturbance Scale for Children (SDSC). Construction and validation of an instrument to evaluate sleep disturbances in childhood and adolescence. J Sleep Res. 1996;5(4):251–61. 10.1111/j.1365-2869.1996.00251.x 9065877

[15] Health Information Standards Organisation. HISO 10001: 2017 Ethnicity data protocols. Ministry of Health Wellington; 2017.

[16] Atkinson J, Salmond C, Crampton P. NZDep2018 Index of deprivation user's manual. Wellington; 2019.

[17] World Health Organisation (2006). WHO Child Growth Standards based on length/height, weight and age. Acta Paediatr Suppl..

[18] Berry RB, Brooks R, Gamaldo CE, Harding SM, Marcus C, Vaughn BV. The AASM manual for the scoring of sleep and associated events. Rules, Terminology and Technical Specifications, Darien, Illinois, American Academy of Sleep Medicine. 2012;176.

[19] White T. Pampro. Cambridge; 2018. 10.5281/zenodo.1187043.

[20] van Hees VT, Fang Z, Langford J, Assah F, Mohammad A, da Silva IC, et al. Autocalibration of accelerometer data for free-living physical activity assessment using local gravity and temperature: an evaluation on four continents. J Appl Physiol. 2014;117(7):738–44. 10.1152/japplphysiol.00421.2014 PMC4187052 25103964

[21] Galland B, Meredith-Jones K, Terrill P, Taylor R. Challenges and Emerging Technologies within the Field of Pediatric Actigraphy. Front Psychiatry. 2014;5:99. 10.3389/fpsyt.2014.00099. eCollection 02014. 10.3389/fpsyt.2014.00099 PMC4139737 25191278

[22] Hyde M, O'driscoll DM, Binette S, Galang C, Tan SK, Verginis N, et al. Validation of actigraphy for determining sleep and wake in children with sleep disordered breathing. J Sleep Res. 2007;16(2):213–6. 10.1111/j.1365-2869.2007.00588.x 17542951

[23] Smith MT, McCrae CS, Cheung J, Martin JL, Harrod CG, Heald JL, et al. Use of Actigraphy for the Evaluation of Sleep Disorders and Circadian Rhythm Sleep-Wake Disorders: An American Academy of Sleep Medicine Systematic Review, Meta-Analysis, and GRADE Assessment. J Clin Sleep Med. 2018;14 (7):1209–30. 10.5664/jcsm.7228 PMC6040804 29991438

[24] Meltzer LJ, Montgomery-Downs HE, Insana SP, Walsh CM. Use of actigraphy for assessment in pediatric sleep research. Sleep Med Rev. 2012;16(5):463–75. 10.1016/j.smrv.2011.10.002 PMC3445439 22424706

[25] Slater JA, Botsis T, Walsh J, King S, Straker LM, Eastwood PR. Assessing sleep using hip and wrist actigraphy. Sleep and Biological Rhythms. 2015;13(2):172–80.

[26] Meltzer LJ, Wong P, Biggs SN, Traylor J, Kim JY, Bhattacharjee R, et al. Validation of Actigraphy in Middle Childhood. Sleep. 2016;39(6):1219–24. 10.5665/sleep.5836 PMC4863209 27091520

[27] Zinkhan M, Berger K, Hense S, Nagel M, Obst A, Koch B, et al. Agreement of different methods for assessing sleep characteristics: a comparison of two actigraphs, wrist and hip placement, and self-report with polysomnography. Sleep medicine. 2014;15(9):1107–14. 10.1016/j.sleep.2014.04.015 25018025

