# Correction: Validation of actigraphy sleep metrics in children aged 8 to 16 years: considerations for device type, placement and algorithms

**DOI:** 10.1186/s12966-024-01608-4

**Published:** 2024-06-08

**Authors:** K. A. Meredith‑Jones, J. J. Haszard, A. Graham‑DeMello, A. Campbell, T. Stewart, B. C. Galland, A. Cox, G. Kennedy, S. Duncan, R. W. Taylor

**Affiliations:** 1 Department of Medicine, University of Otago, PO Box 56, Dunedin, New Zealand; 2 Biostatistics Centre, University of Otago, Dunedin, New Zealand; 3 WellSleep Centre, University of Otago, Wellington, New Zealand; 4 School of Sport and Recreation, Auckland University of Technology, Auckland, New Zealand; 5 Fuzzy Systems Ltd, Dunedin, New Zealand; 6 Department of Women’s and Children’s Health, University of Otago, Dunedin, New Zealand

**Correction: Int J Behav Nutr Phys Act 21, 40 (2024)**

**https://doi.org/10.1186/s12966-024-01590-x**

Following the publication of the Original Article [1], the authors reported errors in reference list, in-text, citations, and the algorithm of Table 5.

Some references were missing, namely:

Acebo C, Sadeh A, Seifer R, Tzischinsky O, Wolfson AR, Hafer A, et al. Estimating sleep patterns with activity monitoring in children and adolescents: how many nights are necessary for reliable measures? Sleep. 1999;22(1):95-103;

Lauderdale DS, Knutson KL, Yan LL, Liu K, Rathouz PJ. Self-reported and measured sleep duration: how similar are they? Epidemiology. 2008;19(6):838-845;

White T. 2018. Pampro. Cambridge, UK. doi:10.5281/zenodo.1187043;

Van Hees VT, Fang Z, Langford J, Assah F, Mohammad A, da Silva IC, et al. Autocalibration of accelerometer data for free-living physical activity assessment using local gravity and temperature: an evaluation on four continents. Journal of Applied Physiology. 2014;117(7):738–744;

Galland B, Meredith-Jones K, Terrill P, Taylor R. Challenges and Emerging Technologies within the Field of Pediatric Actigraphy. Front Psychiatry. 2014;5:99. doi:10.3389/fpsyt.2014.00099. eCollection 02014.

Moreover, two references were irrelevant, namely:

Bunce C. Correlation, agreement, and Bland -Altman analysis: statistical analysis of method comparison studies. Am J Ophthalmol. 2009;148(1):4– 6. 10.1016/j.ajo.2008.09.032 and;

Lee YJ, Lee JY, Cho JH, Choi JH. Interrater reliability of sleep stage scoring: a meta -analysis. J Clin Sleep Med. 2022;18(1):193–202.

Missing references have been added and irrelevant references have been removed.

The correct reference list is thus as follows:

**References**

1. Matricciani L, Paquet C, Galland B, Short M, Olds T. Children's sleep and health: A meta-review. Sleep Med Rev. 2019;46:136-150.(doi):10.1016/j.smrv.2019.1004.1011. Epub 2019 Apr 1023.

2. Acebo C, Sadeh A, Seifer R, Tzischinsky O, Wolfson AR, Hafer A, et al. Estimating sleep patterns with activity monitoring in children and adolescents: how many nights are necessary for reliable measures? Sleep. 1999;22(1):95–103.

3. Lauderdale DS, Knutson KL, Yan LL, Liu K, Rathouz PJ. Self-reported and measured sleep duration: how similar are they? Epidemiology. 2008;19(6):838–845.

4. Barreira TV, Schuna JM, Jr., Mire EF, Katzmarzyk PT, Chaput JP, Leduc G, et al. Identifying children's nocturnal sleep using 24-h waist accelerometry. Med Sci Sports Exerc. 2015;47(5):937–943. doi: 910.1249/MSS.0000000000000486.

5. Cole RJ, Kripke DF, Gruen W, Mullaney DJ, Gillin JC. Automatic sleep/wake identification from wrist activity. Sleep. 1992;15(5):461–469.

6. Galland BC, Kennedy GJ, Mitchell EA, Taylor BJ. Algorithms for using an activity-based accelerometer for identification of infant sleep–wake states during nap studies. Sleep medicine. 2012;13(6):743–751.

7. Sadeh A, Lavie P, Scher A, Tirosh E, Epstein R. Actigraphic home-monitoring sleep-disturbed and control infants and young children: a new method for pediatric assessment of sleep–wake patterns. Pediatrics. 1991;87(4):494–499.

8. Tudor-Locke C, Barreira TV, Schuna JM, Jr., Mire EF, Katzmarzyk PT. Fully automated waist-worn accelerometer algorithm for detecting children's sleep-period time separate from 24-h physical activity or sedentary behaviors. Appl Physiol Nutr Metab. 2014;39(1):53-57. doi: 10.1139/apnm-2013-0173. Epub 2013 Jun 1126.

9. van Hees VT, Sabia S, Jones SE, Wood AR, Anderson KN, Kivimäki M, et al. Estimating sleep parameters using an accelerometer without sleep diary. Scientific Reports. 2018;8(1):12,975.

10. Smith C, Galland B, Taylor R, Meredith-Jones K. ActiGraph GT3X + and Actical Wrist and Hip Worn Accelerometers for Sleep and Wake Indices in Young Children Using an Automated Algorithm: Validation With Polysomnography. Frontiers in psychiatry. 2019;10:958.

11. Quante M, Kaplan ER, Cailler M, Rueschman M, Wang R, Weng J, et al. Actigraphy-based sleep estimation in adolescents and adults: a comparison with polysomnography using two scoring algorithms. Nat Sci Sleep. 2018;10:13-20.(doi):10.2147/NSS.S151085. eCollection 152018.

12. Girschik J, Fritschi L, Heyworth J, Waters F. Validation of Self-Reported Sleep Against Actigraphy. Journal of Epidemiology. 2012;22(5):462–468.

13. Sadeh A, Sharkey KM, Carskadon MA. Activity-based sleep–wake identification: an empirical test of methodological issues. Sleep. 1994;17(3):201–207. doi: 210.1093/sleep/1017.1093.1201.

14. Bruni O, Ottaviano S, Guidetti V, Romoli M, Innocenzi M, Cortesi F, et al. The Sleep Disturbance Scale for Children (SDSC). Construction and validation of an instrument to evaluate sleep disturbances in childhood and adolescence. J Sleep Res. 1996;5(4):251–261.

15. Health Information Standards Organisation. HISO 10001: 2017 Ethnicity data protocols. Ministry of Health Wellington; 2017.

16. Atkinson J, Salmond C, Crampton P. NZDep2018 Index of deprivation user's manual. Wellington, NZ. 2019.

17. World Health Organisation. WHO Child Growth Standards based on length/height, weight and age. Acta Paediatr Suppl. 2006;450:76–85.

18. Berry RB, Brooks R, Gamaldo CE, Harding SM, Marcus C, Vaughn BV. The AASM manual for the scoring of sleep and associated events. Rules, Terminology and Technical Specifications, Darien, Illinois, American Academy of Sleep Medicine. 2012;176.

19. White T. 2018. Pampro. Cambridge, UK. doi:10.5281/zenodo.1187043

20. van Hees VT, Fang Z, Langford J, Assah F, Mohammad A, da Silva IC, et al. Autocalibration of accelerometer data for free-living physical activity assessment using local gravity and temperature: an evaluation on four continents. Journal of Applied Physiology. 2014;117(7):738–744.

21. Galland B, Meredith-Jones K, Terrill P, Taylor R. Challenges and Emerging Technologies within the Field of Pediatric Actigraphy. Front Psychiatry. 2014;5:99.doi:10.3389/fpsyt.2014.00099. eCollection 02014.

22. Hyde M, O'driscoll DM, Binette S, Galang C, Tan SK, Verginis N, et al. Validation of actigraphy for determining sleep and wake in children with sleep disordered breathing. Journal of Sleep Research. 2007;16(2):213–216.

23. Smith MT, McCrae CS, Cheung J, Martin JL, Harrod CG, Heald JL, et al. Use of Actigraphy for the Evaluation of Sleep Disorders and Circadian Rhythm Sleep–Wake Disorders: An American Academy of Sleep Medicine Systematic Review, Meta-Analysis, and GRADE Assessment.. Journal of Clinical Sleep Medicine. 2018;14 (7):1209–1230.

24. Meltzer LJ, Montgomery-Downs HE, Insana SP, Walsh CM. Use of actigraphy for assessment in pediatric sleep research. Sleep medicine reviews. 2012;16(5):463–475.

25. Slater JA, Botsis T, Walsh J, King S, Straker LM, Eastwood PR. Assessing sleep using hip and wrist actigraphy. Sleep and Biological Rhythms. 2015;13(2):172–180.

26. Meltzer LJ, Wong P, Biggs SN, Traylor J, Kim JY, Bhattacharjee R, et al. Validation of Actigraphy in Middle Childhood. Sleep. 2016;39(6):1219–1224.

27. Zinkhan M, Berger K, Hense S, Nagel M, Obst A, Koch B, et al. Agreement of different methods for assessing sleep characteristics: a comparison of two actigraphs, wrist and hip placement, and self-report with polysomnography. Sleep medicine. 2014;15(9):1107–1114.

In-text citations correspond to the new references and some have been adjusted to align with the correct references:

**Table:** 

Page	Incorrect / missing citation	Correct citation
6	HDCZA	HDCZA [9]
24	The findings from this much larger and more comprehensive study are broadly consistent with the original validation studies and a review of previous validation studies in children, which show that accuracy (0.84–0.92) and sensitivity (0.82–0.96) are generally good, whereas specificity (0.20–0.65) is considerably lower [20]. However it is clear from both previous research and the current study that the specificity (54–77%) [20], or ability to detect periods of wakefulness in the sleep period window, of most algorithms was better when the device was worn at the wrist, with estimates ranging from 67 to 90%. These figures are considerably higher than those observed in adult studies, which have reported specificities of 34–46% for the HDCZA, Sadeh and Cole algorithms when validated in adult samples [9, 11, 21]. These discrepancies may arise because of differences in sleep characteristics between children and adults. In our study, most children had long periods of sleep without wakefulness during the night. Although immobility generally infers sleep in accelerometery-based assessment, immobility is possible during periods of wakefulness and as such can be mistakenly identified as sleep by actigraphy; it is likely this occurs more in adults because they have more periods of conscious nocturnal awakenings than children [11, 19]	The findings from this much larger and more comprehensive study are broadly consistent with the original validation studies and a review of previous validation studies in children, which show that accuracy (0.84–0.92) and sensitivity (0.82–0.96) are generally good, whereas specificity (0.20–0.65) is considerably lower [24]. However it is clear from both previous research and the current study that the specificity (54–77%) [24], or ability to detect periods of wakefulness in the sleep period window, of most algorithms was better when the device was worn at the wrist, with estimates ranging from 67 to 90%. These figures are considerably higher than those observed in adult studies, which have reported specificities of 34–46% for the HDCZA, Sadeh and Cole algorithms when validated in adult samples [9, 11, 25]. These discrepancies may arise because of differences in sleep characteristics between children and adults. In our study, most children had long periods of sleep without wakefulness during the night. Although immobility generally infers sleep in accelerometery-based assessment, immobility is possible during periods of wakefulness and as such can be mistakenly identified as sleep by actigraphy; it is likely this occurs more in adults because they have more periods of conscious nocturnal awakenings than children [11, 26]
25	The wrist placement was also superior to the thigh, lower back and hip for estimates of sleep onset, offset, quantity (TST and SPT) and WASO for most algorithms. Prior research has also indicated that hipworn accelerometers tend to overestimate total sleep time and sleep efficiency while underestimating wake after sleep onset (WASO), resulting in lower specificity compared to wrist-worn devices [21, 24]	The wrist placement was also superior to the thigh, lower back and hip for estimates of sleep onset, offset, quantity (TST and SPT) and WASO for most algorithms. Prior research has also indicated that hipworn accelerometers tend to overestimate total sleep time and sleep efficiency while underestimating wake after sleep onset (WASO), resulting in lower specificity compared to wrist-worn devices [25, 27]

Moreover, Table 5 of the Original Article mistakenly mentioned "Van Hees" in the Algorithm section. The correct algorithm is "HDCZA".

The original article has been corrected.
