# Supplementary material for: Validation of actigraphy sleep metrics in children aged 8 to 16 years: considerations for device type, placement and algorithms
Source: Int J Behav Nutr Phys Act. 2024 Apr 16;21:40. doi: 10.1186/s12966-024-01590-x (PMC11020269; doi:10.1186/s12966-024-01590-x)
Supplement: Supplementary file 1 — Additional file 1: Supplementary Table 1. Number of participants with missing data from each algorithm (n=131). Supplementary Table 2. Sensitivity analysis for sensitivity, specificity, and accuracy of epoch-by-epoch comparisons with PSG for sleep with half PSG epochs assigned as sleep (rather than wake). [file 12966_2024_1590_MOESM1_ESM.docx]

**Supplementary Table 1.** Number of participants with missing data from each algorithm (n=131)

|  | Actigraph GT3x | | AX3 | | |
| --- | --- | --- | --- | --- | --- |
|  | Hip | Wrist | Thigh | Back | Wrist |
| Count-scaled | 0 | 1 | 2 | 0 | 0 |
| Sadeh 1 | 0 | 1 | 2 | 0 | 0 |
| Sadeh 2 | 4 | 3 | 4 | 7 | 2 |
| Cole-Kripke 1 | 0 | 0 | 2 | 0 | 1 |
| Cole-Kripke 2 | 0 | 1 | 2 | 0 | 0 |
| Cole-Kripke 3 | 6 | 4 | 9 | 8 | 1 |
| Tudor-Locke 1 | 9 | 2 | 4 | 7 | 0 |
| Tudor-Locke 2 | 2 | 4 | 7 | 4 | 2 |
| Tudor-Locke 3 | 2 | 2 | 3 | 6 | 2 |
| Tudor-Locke 4 | 2 | 3 | 5 | 5 | 1 |
| HDCZA | 4 | 4 | 3 | 5 | 2 |
| No missing data | 118 | 125 | 118 | 119 | 128 |

**Supplementary Table 2**. Sensitivity analysis for sensitivity, specificity, and accuracy of epoch-by-epoch comparisons with PSG for sleep with half PSG epochs assigned as sleep (rather than wake)

| Device | Placement | Algorithm | Mean accuracy  % (95% CI) | Mean sensitivity  % (95% CI) | Mean specificity  % (95% CI) |
| --- | --- | --- | --- | --- | --- |
| Actigraph GT3x | Hip | Count-scaled | 78.0 (76.2, 79.9) | 93.1 (91.8, 94.4) | 57.8 (54.6, 60.9) |
|  |  | Sadeh 1 | 80.9 (78.9, 82.9) | 97.0 (95.6, 98.2) | 59.3 (55.9, 62.6) |
|  |  | Sadeh 2 | 81.2 (79.2, 83.3) | 96.9 (95.5, 98.3) | 59.9 (56.5, 63.4) |
|  |  | Cole-Kripke 1 | 83.3 (81.2, 85.4) | 92.9 (91.2, 94.6) | 70.9 (67.4, 74.5) |
|  |  | Cole-Kripke 2 | 76.0 (74.1, 77.9) | 98.5 (97.4, 99.6) | 45.4 (42.5, 48.3) |
|  |  | Cole-Kripke 3 | 76.4 (74.4, 78.3) | 98.5 (97.3, 99.6) | 46.3 (43.4, 49.2) |
|  |  | Tudor-Locke 1 | 76.6 (74.6, 78.5) | 98.5 (97.4, 99.7) | 46.2 (43.2, 49.1) |
|  |  | Tudor-Locke 2 | 75.8 (73.9, 77.7) | 98.6 (97.5, 99.7) | 45.0 (42.0, 47.9) |
|  |  | Tudor-Locke 3 | 80.7 (78.7, 82.8) | 97.3 (96.0, 98.6) | 58.1 (54.7, 61.5) |
|  |  | Tudor-Locke 4 | 81.0 (79.1, 82.9) | 97.3 (96.0, 98.6) | 58.3 (54.9, 61.6) |
|  |  | HDCZA | 85.8 (83.6, 88.1) | 84.1 (81.3, 87.0) | 89.3 (87.1, 91.5) |
|  | Wrist | Count-scaled | 81.7 (79.7, 83.7) | 90.2 (88.7, 91.8) | 70.5 (67.1, 73.9) |
|  |  | Sadeh 1 | 84.0 (82.0, 86.0) | 88.5 (86.7, 90.4) | 78.4 (75.0, 81.8) |
|  |  | Sadeh 2 | 84.3 (82.4, 86.3) | 88.6 (86.7, 90.4) | 79.0 (75.7, 82.4) |
|  |  | Cole-Kripke 1 | 78.6 (76.6, 80.6) | 75.3 (73.1, 77.5) | 83.9 (80.7, 87.1) |
|  |  | Cole-Kripke 2 | 84.6 (82.6, 86.7) | 92.7 (90.9, 94.4) | 74.2 (70.6, 77.7) |
|  |  | Cole-Kripke 3 | 85.3 (83.5, 87.2) | 93.1 (91.8, 94.5) | 75.0 (71.5, 78.5) |
|  |  | Tudor-Locke 1 | 84.5 (82.4, 86.5) | 93.4 (91.7, 95.1) | 72.6 (69.1, 76.1) |
|  |  | Tudor-Locke 2 | 85.0 (83.1, 86.9) | 93.3 (91.6, 95.0) | 73.3 (69.9, 76.7) |
|  |  | Tudor-Locke 3 | 84.0 (82.0, 86.0) | 90.1 (88.3, 91.9) | 76.1 (72.8, 79.5) |
|  |  | Tudor-Locke 4 | 84.2 (82.2, 86.2) | 90.1 (88.3, 91.9) | 76.4 (73.1, 79.8) |
|  |  | HDCZA | 86.2 (84.1, 88.2) | 83.6 (81.3, 86.0) | 90.6 (88.4, 92.8) |
| Axivity | Back | Count-scaled | 76.7 (74.5, 78.8) | 92.0 (90.4, 93.6) | 56.0 (52.3, 59.6) |
|  |  | Sadeh 1 | 78.1 (75.9, 80.4) | 95.2 (93.5, 96.9) | 55.4 (51.8, 59.1) |
|  |  | Sadeh 2 | 79.2 (77.0, 81.5) | 95.6 (94.1, 97.1) | 57.4 (53.8, 60.9) |
|  |  | Cole-Kripke 1 | 81.1 (78.8, 83.4) | 92.3 (90.5, 94.2) | 66.7 (62.8, 70.6) |
|  |  | Cole-Kripke 2 | 74.5 (72.4, 76.6) | 96.9 (95.4, 98.3) | 44.1 (40.9, 47.3) |
|  |  | Cole-Kripke 3 | 75.3 (73.3, 77.4) | 97.2 (95.9, 98.4) | 45.6 (42.5, 48.7) |
|  |  | Tudor-Locke 1 | 75.3 (73.2, 77.3) | 97.2 (96.0, 98.4) | 45.3 (42.1, 48.4) |
|  |  | Tudor-Locke 2 | 75.0 (73.0, 77.3) | 97.4 (96.2, 98.6) | 44.7 (41.5, 47.9) |
|  |  | Tudor-Locke 3 | 78.7 (76.3, 81.2) | 95.1 (93.3, 96.8) | 56.9 (53.3, 60.4) |
|  |  | Tudor-Locke 4 | 79.0 (76.8, 81.1) | 95.7 (94.3, 97.2) | 56.9 (53.3, 60.4) |
|  |  | HDCZA | 86.4 (84.0, 88.8) | 86.1 (84.0, 88.3) | 90.9 (88.8, 93.0) |
|  | Thigh | Count-scaled | 74.0 (71.8, 76.2) | 87.8 (85.8, 89.7) | 55.7 (52.5, 58.8) |
|  |  | Sadeh 1 | 78.0 (77.4, 82.5) | 90.3 (87.8, 92.8) | 66.3 (62.5, 70.0) |
|  |  | Sadeh 2 | 80.3 (77.8, 82.8) | 90.7 (88.3, 93.1) | 66.6 (62.8, 70.3) |
|  |  | Cole-Kripke 1 | 80.2 (77.6, 82.7) | 84.0 (81.3, 86.7) | 75.5 (71.6, 71.3) |
|  |  | Cole-Kripke 2 | 77.7 (75.2, 80.1) | 93.9 (91.7, 96.1) | 56.1 (52.7, 59.5) |
|  |  | Cole-Kripke 3 | 78.4 (76.0, 80.8) | 94.1 (91.9, 96.3) | 57.6 (54.2, 60.9) |
|  |  | Tudor-Locke 1 | 77.8 (75.4, 80.3) | 93.8 (91.6, 96.1) | 56.5 (53.2, 59.9) |
|  |  | Tudor-Locke 2 | 79.0 (76.8, 81.1) | 95.4 (93.9, 96.8) | 57.3 (54.0, 60.7) |
|  |  | Tudor-Locke 3 | 79.9 (77.3, 82.5) | 90.3 (87.8, 92.8) | 66.2 (62.4, 70.0) |
|  |  | Tudor-Locke 4 | 81.1 (78.7, 83.4) | 91.4 (89.3, 93.4) | 67.4 (63.7, 71.1) |
|  |  | HDCZA | 87.8 (86.0, 89.6) | 86.9 (85.4, 88.4) | 91.0 (88.8, 93.1) |
|  | Wrist | Count-scaled | 79.2 (76.8, 81.6) | 85.6 (83.2, 87.9) | 70.9 (67.3, 74.5) |
|  |  | Sadeh 1 | 81.3 (78.8, 83.9) | 83.5 (80.9, 86.0) | 76.9 (76.0, 83.2) |
|  |  | Sadeh 2 | 81.8 (79.4, 84.3) | 84.1 (81.7, 86.4) | 80.1 (76.5, 83.7) |
|  |  | Cole-Kripke 1 | 76.4 (71.4, 78.7) | 71.8 (69.6, 74.1) | 84.0 (80.6, 87.5) |
|  |  | Cole-Kripke 2 | 81.9 (79.4, 84.4) | 87.3 (84.7, 90.0) | 75.0 (71.2, 78.8) |
|  |  | Cole-Kripke 3 | 82.0 (79.5, 84.5) | 87.3 (84.6, 90.0) | 75.3 (71.6, 79.1) |
|  |  | Tudor-Locke 1 | 81.9 (79.4, 84.4) | 87.3 (84.7, 90.0) | 75.0 (71.2, 78.8) |
|  |  | Tudor-Locke 2 | 82.6 (80.2, 84.9) | 88.5 (86.4, 90.6) | 75.1 (71.2, 78.9) |
|  |  | Tudor-Locke 3 | 81.8 (79.4, 84.3) | 84.1 (81.7, 86.4) | 80.1 (76.5, 83.7) |
|  |  | Tudor-Locke 4 | 81.6 (79.2, 84.0) | 84.0 (81.8, 86.3) | 79.6 (75.9, 83.2) |
|  |  | HDCZA | 87.8 (85.8, 89.7) | 85.7 (84.1, 87.3) | 93.1 (91.3, 94.9) |
